# Role of mitochondria and cardiolipins in growth inhibition of breast cancer cells by retinoic acid

**DOI:** 10.1186/s13046-019-1438-y

**Published:** 2019-10-29

**Authors:** Mineko Terao, Laura Goracci, Valentina Celestini, Mami Kurosaki, Marco Bolis, Alessandra Di Veroli, Arianna Vallerga, Maddalena Fratelli, Monica Lupi, Alessandro Corbelli, Fabio Fiordaliso, Maurizio Gianni, Gabriela Paroni, Adriana Zanetti, Gabriele Cruciani, Enrico Garattini

**Affiliations:** 1 Laboratory of Molecular Biology, Istituto di Ricerche Farmacologiche Mario Negri IRCCS, via La Masa 19, 20156 Milan, Italy; 2 Department of Chemistry, Biology and Biotechnology, University of Perugia, via Elce di Sotto 8, 06123 Perugia, Italy; 3 Consortium for Computational Molecular and Materials Sciences (CMS), via Elce di Sotto 8, 06123 Perugia, Italy; 4 Department of Oncology, Istituto di Ricerche Farmacologiche Mario Negri IRCCS, via La Masa 19, 20156 Milan, Italy; 5 Department of Cardiovascular Research, Istituto di Ricerche Farmacologiche Mario Negri IRCCS, via La Masa 19, 20156 Milan, Italy

**Keywords:** Retinoic acid, Breast cancer, Lipidomics, Oxidative phosphorylation

## Abstract

**Background:**

All-trans-retinoic-acid (ATRA) is a promising agent in the prevention/treatment of breast-cancer. There is growing evidence that reprogramming of cellular lipid metabolism contributes to malignant transformation and progression. Lipid metabolism is implicated in cell differentiation and metastatic colonization and it is involved in the mechanisms of sensitivity/resistance to different anti-tumor agents. The role played by lipids in the anti-tumor activity of ATRA has never been studied.

**Methods:**

We used 16 breast cancer cell-lines whose degree of sensitivity to the anti-proliferative action of ATRA is known. We implemented a non-oriented mass-spectrometry based approach to define the lipidomic profiles of each cell-line grown under basal conditions and following treatment with ATRA. To complement the lipidomic data, untreated and retinoid treated cell-lines were also subjected to RNA-sequencing to define the perturbations afforded by ATRA on the whole-genome gene-expression profiles. The number and functional activity of mitochondria were determined in selected ATRA-sensitive and –resistant cell-lines. Bio-computing approaches were used to analyse the high-throughput lipidomic and transcriptomic data.

**Results:**

ATRA perturbs the homeostasis of numerous lipids and the most relevant effects are observed on cardiolipins, which are located in the mitochondrial inner membranes and play a role in oxidative-phosphorylation. ATRA reduces the amounts of cardiolipins and the effect is associated with the growth-inhibitory activity of the retinoid. Down-regulation of cardiolipins is due to a reduction of mitochondria, which is caused by an ATRA-dependent decrease in the expression of nuclear genes encoding mitochondrial proteins. This demonstrates that ATRA anti-tumor activity is due to a decrease in the amounts of mitochondria causing deficits in the respiration/energy-balance of breast-cancer cells.

**Conclusions:**

The observation that ATRA anti-proliferative activity is caused by a reduction in the respiration and energy balance of the tumor cells has important ramifications for the therapeutic action of ATRA in breast cancer. The study may open the way to the development of rational therapeutic combinations based on the use of ATRA and anti-tumor agents targeting the mitochondria.

## Background

All-trans-retinoic acid (ATRA) is a non-conventional and promising therapeutic agent acting on different types of solid/hematologic malignancies [1–5] and it is used in the treatment of acute-promyelocytic-leukemia (APL) [6]. In APL patients, ATRA induces the differentiation of leukemic cells, which is at the basis of its therapeutic activity. The unusual mechanism of action and the available pre-clinical data have raised interest in ATRA for the treatment of breast-cancer [7, 8].

Breast-cancer is a heterogeneous disease [9], although it is traditionally classified in three subgroups according to the presence/absence of the estrogen-receptor (ER), the progesterone-receptor (PR) and the HER2 protein. In addition, breast-cancers can be divided into luminal and basal tumors according to the morphological characteristics. Steroid hormone receptors are considered to be good therapeutic targets in luminal breast-cancer [10]. Recently, we demonstrated that a large proportion of luminal and ER^+^ mammary tumors are characterized by sensitivity to the anti-proliferative action of ATRA, while the triple-negative counterparts tend to be resistant [11, 12]. The biological activity of ATRA is mediated by specific steroid receptors (RARα, RARβ and RARγ), which act as ligand-dependent transcription factors under the form of heterodimers with other retinoid receptors known as RXRs (RXRα, RXRβ and RXRγ) [7, 13]. ATRA is a pan-RAR agonist, binding all RARs with the same affinity. In breast-cancer, we identified RARα as the retinoid receptor mediating the growth-inhibitory activity exerted by ATRA [11].

There is growing evidence that reprogramming of cellular lipid metabolism contributes to malignant transformation and progression [14–18]. In addition, lipids play a role in the mechanisms of sensitivity/resistance to different anti-tumor agents [19–22]. The involvement of lipids in the anti-tumor activity of ATRA has never been studied and this type of studies is now facilitated by the availability of technologies allowing the definition of the cellular lipidomic profiles [23–25]. The potential relevance of lipids for the anti-tumor action of ATRA is of interest given the role played in the growth, differentiation and metastatic spread, three processes affected by the retinoid [11, 26, 27].

Here, we evaluate the constitutive lipidomic profiles of breast-cancer cell-lines recapitulating the heterogeneity of the disease [11, 12] and the perturbations induced by ATRA. The lipidomic profiles of luminal and basal breast-cancer cell-lines are distinct. In addition, we identify cardiolipins (*CLs*) as the main lipid class modulated by ATRA in retinoid-sensitive breast-cancer cell-lines. Mechanistic and RNA-sequencing studies show that the ATRA-dependent down-regulation of *CLs* in sensitive cell-lines is accompanied by a reduction in the amounts and activity of mitochondria.

## Materials and methods

### Cell-lines

The source and the characteristics of the 16 breast-cancer cell-lines used are available in Additional file 1. The generation of the RARα over-expressing (*RARA-C5*) and relative control (*Vect-C1*) clones from *MDA-MB-453* cells as well as the RARα silenced (*RARsA-sh18*) and relative control (*Vect-C6*) clones from *SK-BR-3* cells have been described [11]. The growth of cells was determined with the sulforhodamine assay [11].

### Single-cell motility

Single-cell motility assays were performed on BSA-coated culture wells by time-lapse microscopy, using the Imaging Station CellRˆ (Olympus, Segrate, Italy) and the software Image J (Rasband W, National Institutes of Health, Bethesda, MD).

### Untargeted lipidomics

Untargeted lipidomics studies were performed with Lipostar, a high-throughput software supporting targeted and untargeted liquid-chromatography/mass-spectrometry (LC-MS) lipidomics [23, 28]. Further details on the methodological approach can be found in Additional file 1.

### Mitochondrial studies

Mitochondria were stained with MitoTarcker Deep Red FM (Invitrogen) according to the manufacturer instructions. Following staining, cells were fixed with 2% formalin and subjected to quantitative FACS analysis, using a fluorescence activated cell sorter (FACS, Becton and Dickinson). In the case of the experiments performed on the *SK-BR-3*, *HCC-1419*, *MDA-MB-361* and *HCC-202* lines, mitotracker stained cells were also subjected to quantitative microscopic analysis using the ImageJ software. Cells were counterstained with Hoechst 3342 (Thermofisher) for the determination of cell nuclei. For each experimental point, a minimum of 200 cells/field in at least 4 fields/experimental triplicate were quantitated. Mitochondria were isolated using a described protocol [29]. The enzymatic activity of mitochondrial complexes was determined on isolated mitochondria [30, 31]. The microviscosity of mitochondrial membranes was measured as described by M. Salmona et al. following staining with 1,6-diphenyl-1,3,5-hexatriene as a fluorescent probe [32].

### RNA-sequencing studies

Three paired biological replicates of each breast-cancer cell-line were grown in DMEMF12 medium containing 5% charcolated FBS (Fetal Bovine Serum, Gibco) for 24 h. Cells were treated with vehicle (DMSO) or ATRA (10^− 6^ M) for another 24 h. RNA was extracted with the mRNeasy Mini Kit (QIAGEN). RNA sequencing was performed using the Illumina TruSeq RNA library-preparation kit and sequenced on the Illumina NextSeq500 with paired-end, 150 base pair long reads. The overall quality of sequencing reads was evaluated using FastQC [33]. Sequence alignments to the reference human genome (GRCh38) were performed using STAR (v.2.5.2a). Gene-expression was quantified using the comprehensive annotations available in *Gencode* [34]. Specifically, we used the *v27* release of the Gene Transfer File (GTF). Raw-counts were further processed in the *R* Statistical environment and downstream differential expression analysis was performed using the *DESeq2* pipeline. All the RNA-sequencing data relevant for this study were deposited in the EMBL-EBI Arrayexpress database (Accession No: E-MTAB-8408). Genes characterized by low mean normalized counts were filtered out by the *Independent Filtering* feature embedded in *DESeq2* (alpha = 0.05). *DESeq2*-computed statistics were used as input for gene-set enrichment testing performed with the pre-ranked version of Camera. Statistical enrichments were determined for gene-sets obtained from the Hallmark (H), which are curated by the Molecular Signature DataBase (*MSigDB*).

### Transmission electron microscopy

ATRA-sensitive *SK-BR-3* and ATRA-resistant *HCC-1419* cell lines were grown on plastic Petri dishes and fixed in phosphate buffer 0.12 M pH 7.4 containing 4% paraformaldehyde and 2% glutaraldehyde (Electron Microscopy Sciences, code #16220) for 10 min. Cells were scraped from the dishes, centrifuged at 13,000 rpm and kept in 0.12 M phosphate buffer containing 4% paraformaldehyde and 2% glutaraldehyde for 2 h. After post-fixation in 0.12 M cacodylate buffer containing 1% OsO_4_ for 2 h and subsequent dehydration in graded series of ethanol, samples were cleared in propylene oxide, embedded in Epoxy medium (Epon 812 Fluka) and polymerized at 60 °C for 72 h. Ultrathin sections (70 nm thickness) were obtained with a Leica EM UC6 ultramicrotome, counterstained with uranyl acetate and lead citrate and examined with an Energy Filter Transmission Electron Microscope (EFTEM, ZEISS LIBRA® 120) equipped with a yttrium aluminium garnet (YAG) scintillator slow-scan charge-coupled device (CCD) camera (Sharp eye, TRS, Moorenweis, Germany). The numerical density of mitochondria (N_V_, n/μm^3^) was estimated by morphometrical analysis using 30 digitized electron microscope fields of cells for each group acquired by the iTem software (Olympus Soft Imaging Solutions, Germany) and digitally superimposing an orthogonal grid with ImageJ (1.52a version). Briefly, the mitochondrial profile area density (N_A_) was estimated by the ratio between the number of mitochondria and the cytoplasmic area. Mitochondrial volume density (V_V_) was determined by the ratio of grid points falling over mitochondria divided by the total number of points of the grid contained in the cytoplasm. The numerical density of mitochondria (N_V_) was then estimated for each cell using the formula: N_V_ = (1/β) (N_A_^3/2^ / V_V_^1/2^), where β is the shape coefficient for ellipsoidal mitochondria, calculated from the ratio of the armonic mean of major and minor axis of mitochondria sections measured on digital images. The mean mitochondrial volume was calculated for each cell as the ratio of mitochondrial volume density V_V_ and numerical density N_V_.

### Measurement of mitochondrial membrane microviscosity

The microviscosity of mitochondrial membranes was measured as described by M. Salmona et al. following staining with 1,6-diphenyl-1,3,5-hexatriene as a fluorescent probe [3]. Isolated mitochondria containing an equal amount of proteins were incubated with 1,6-diphenyl-1,3,5-hexatriene (2 × 10^− 6^ M) for 30 min at 37° and the fluorescence polarization values were determined using a fluorescence detector (Infinite F500, TECAN, Switzerland). The fluorescence polarization (FP) value is a function of the emission value (emission = 420 nm), which was detected through analyzers oriented in parallel (FP1) and perpendicular (FP2) to the direction of polarization of the excitation beam (excitation = 365 nm), according to the eq. FP = (FP2 - FP1 / FP2 + FP1).

## Results

### ATRA sensitivity and constitutive lipidomic profiles in luminal and basal breast-cancer cell-lines

**Fig. 1:**
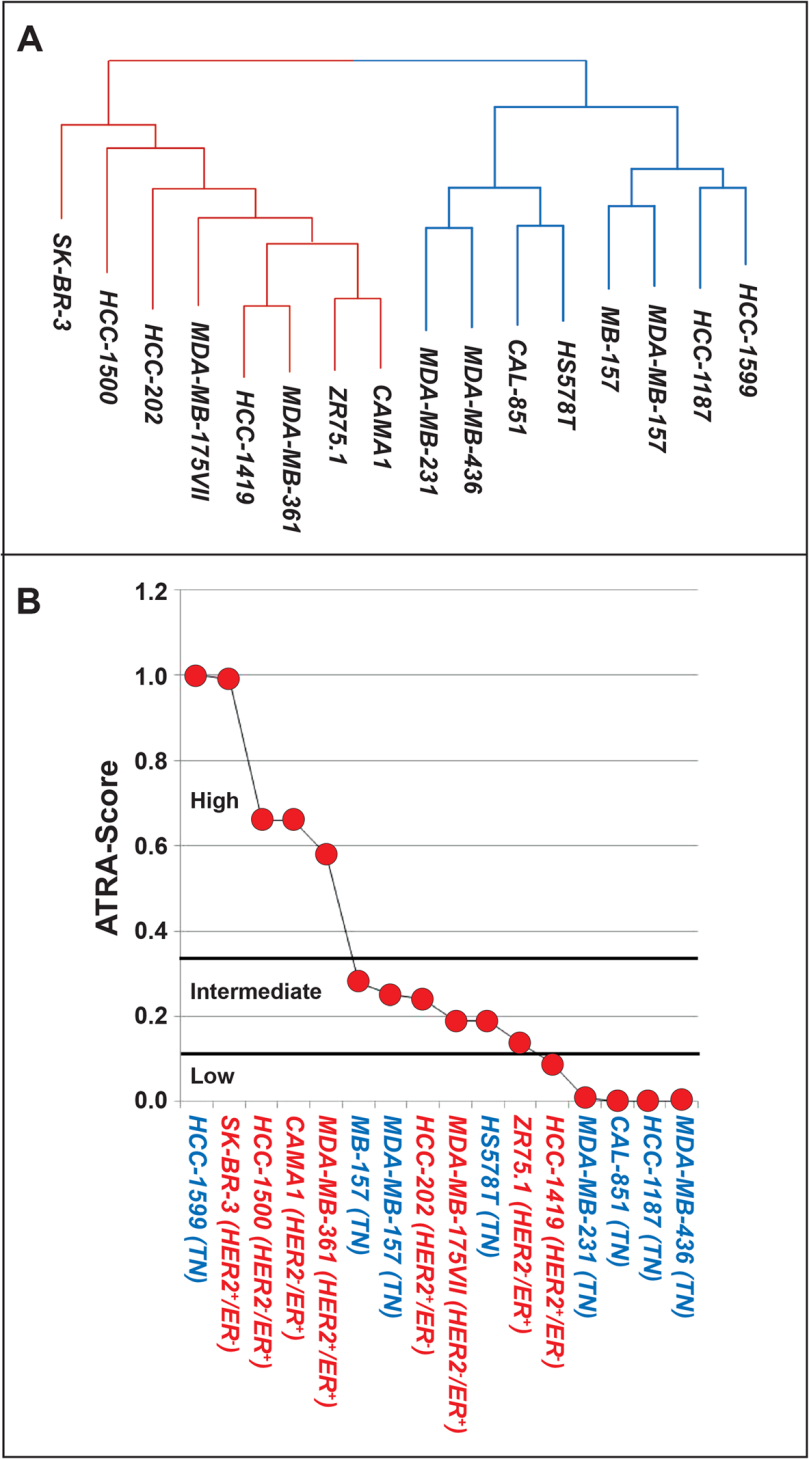
Characteristics and sensitivity to ATRA of the breast cancer cell-lines used in the study. **a** The panel illustrates a dendrogram of the breast cancer cell-lines used in the study. Clustering of the cell-lines is based on the basal gene-expression profiles determined for each cell-line by NGS (Next Generation Sequencing). **b** The panel illustrates the sensitivity of each cell-line to the anti-proliferative action of ATRA defined by application of the *ATRA-score* model. The higher is the *ATRA-score* value the higher is the sensitivity of the cell-line to ATRA. The horizontal lines indicate the ATRA-score threshold values used to define the cell-lines characterized by high, intermediate and low sensitivity to ATRA. The cell-lines marked in blue are characterized by a basal phenotype, while the ones marked in red are endowed with a luminal phenotype. ER = Estrogen receptor; HER2 = Human epidermal growth factor receptor 2; TN = Triple negative

To conduct the study, we used 16 cell-lines whose degree of sensitivity to the anti-proliferative action of ATRA has been determined [11, 12]. Eight cell-lines come from triple-negative (*TN*) breast-cancers, while the other 8 cell-lines derive from luminal tumors characterized for HER2 and ER/PR expression. Consistent with the tumor origin, the constitutive gene-expression profiles determined by RNA-sequencing classify the cell-lines in two distinct basal and luminal groups (Fig. 1a). The 16 cell-lines are ranked according to their quantitative response to the anti-proliferative effect of ATRA using the continuous *ATRA-score* index (Fig. 1b) [11]. One basal (*HCC-1599*) and 4 luminal (*SK-BR-3, HCC-1500, CAMA1* and *MDA-MB-361*) cell-lines are classified as highly sensitive to ATRA. Three luminal (*HCC-202; MDA-MB-175VII; ZR75.1*) and 3 basal (*MB-157; MDA-MB-157; HS578T*) cell-lines are endowed with intermediate sensitivity. One luminal (*HCC-1419*) and 4 basal (*MDA-MB-231; CAL-851; HCC-1187; MDA-MB-436*) cell-lines show low sensitivity/resistance to ATRA.

**Fig. 2:**
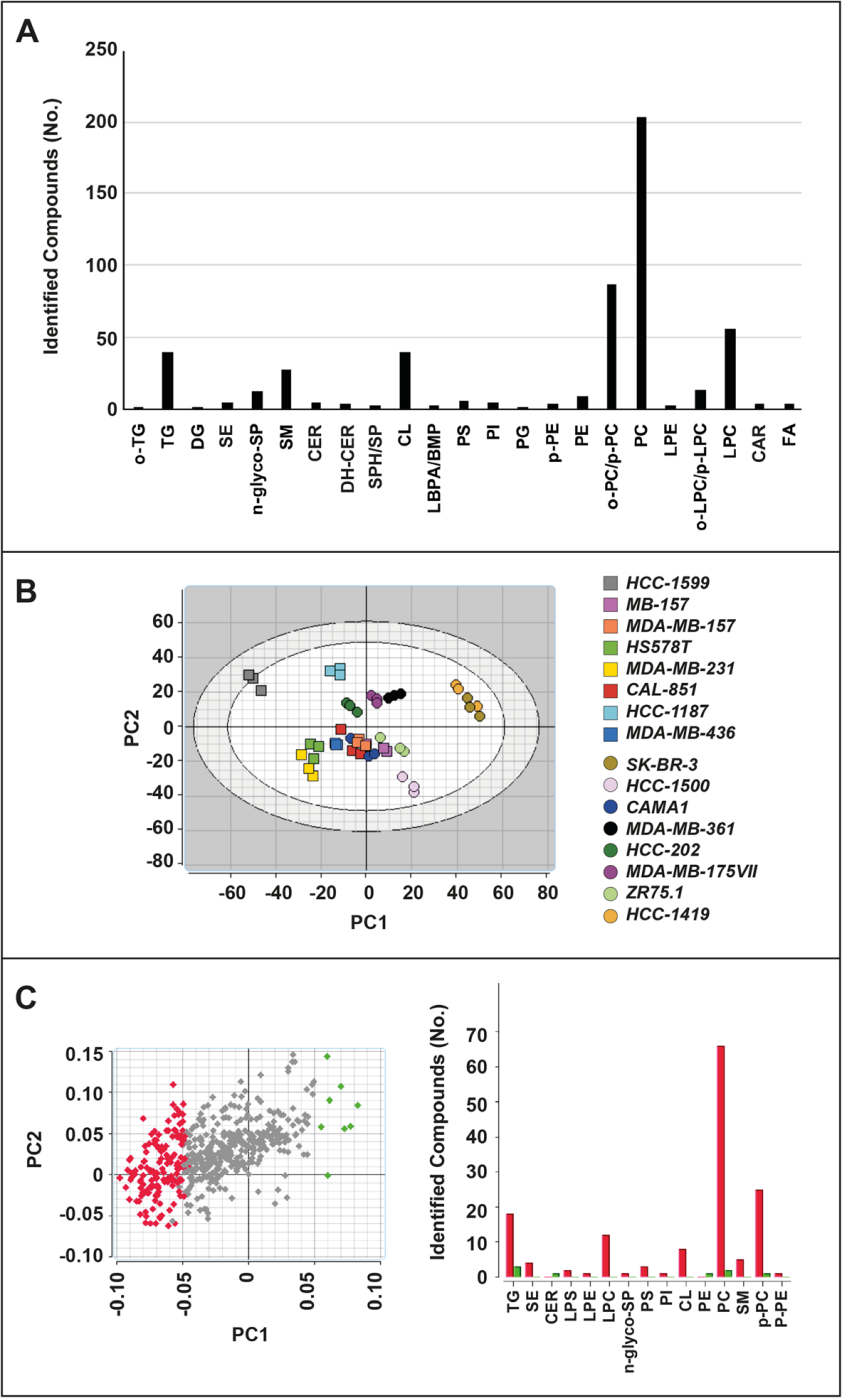
Basal lipidomic profiles of breast cancer cells. **a** The panel illustrates the complement of constitutive lipids determined in breast cancer cells growing under standard conditions. Lipids are classified in the indicated groups on the basis of their general chemical structure. The number of chemical species identified for each class of lipids are indicated on the vertical axis. **b** The diagram shows a bidimensional principal component analysis (PCA) of the constitutive lipidomic profiles determined in each cell-line exposed to DMSO. **c** The left PCA analysis illustrates the lipid species whose levels are significantly higher in luminal than basal (green points) and basal than luminal (red points) cell-lines. The right bar graph indicates the lipid classes whose levels are significantly higher in luminal (green bars) and basal (red bars) cell-lines. The data are expressed as in panel (A). *o-TG/p-TG* = alkyldiacylglycerols/1Z-alkenyldiacylglycerols; *TG* = triacylglycerols; *DG* = diacylglycerols; *SE* = steryl esters; *N-glyco-SP* = neutral glycosphingolipids; *SM* = sphingomyelins; *CER* = ceramides; *DHCER* = dihydroceramides; *SPH/SP* = sphingosines/sphinganines; *CL* = cardiolipins; *LBPA/BMP* = lysobisphosphatidic acid/ bis(monoacylglycero)phosphate; *PS* = phosphatidylserines; *PI* = phosphatidylinositols; *PG* = phosphatidylglycerol; *p-PE* = 1-alkyl-2-acylglycerophosphoethanolamines/1-alkenyl-2-acylglycerophosphoethanolamines; *PE* = phosphatidylethanolamines; *o-PC/p-PC* = 1-alkyl-2-acylglycerophosphocholines/1-alkenyl-2-acylglycerophosphocholines; *PC* = phosphatidylcholines; *LPE* = lysophosphatidylethanolamines; o-LPC/p-LPC = 1-alkyl-glycerophosphocholines/1-alkenyl-glycerophosphocholines; *LPC* = lysophosphatidylcholines; *CAR* = acylcarnitines; *FA* = fatty acids

We used a LC/MS-based approach [23] to define the lipidomic profiles of each cell-line grown under basal conditions. We generated a fingerprint composed of 530 chemical features identified as lipid species, some of them in multiple adduct forms. Lipid species were grouped in 23 classes according to their chemical structures. Each class contains a different number of chemical species (Fig. 2a and Additional file 2: Table S1). For instance, Diacylglycerophosphocholines (*PCs*) consist of 203 features, while 40 cardiolipins (*CLs*) are identified. PCA of the constitutive lipid profiles separates luminal from basal cell-lines (Fig. 2b). Thus, the two cell-line groups are characterized not only by different gene-expression patterns, but also by different complements of lipids. Compared to the luminal counterparts, basal cell-lines show greater amounts of many *TGs*, diacylglycerophosphoserines or phosphatidylserines (*PSs*) and *PCs*, some diacylglycerophosphoinositols or phosphatidylinositols (*PIs*), neutral glycosphingolipids (*N-glyco-SPs*), *CLs*, lysophosphatidylcholines (*LPCs*), sphingomyelins (*SMs*) and 1-alkyl-2-acylglycerophosphocholines/1-alkenyl-2-acylglycerophosphocholines (o*-PCs/ p-PCs*), as well as all the identified steryl-esters (*SEs*), monoacylglycerophosphoethanolamines (lysophosphatidylethanolamines, *LPEs*) and 1-alkenyl-phosphatidylethanolamine (*p-PE*) (Fig. 2c). In contrast, the levels of 3 triacylglycerols (*TGs*), 2 *PCs*, 1 *p-PC*, 1 ceramide (*CER*) and 1 diacylglycerophosphoethanolamine (phosphatidylethanolamine, *PE*) are higher in luminal than basal cell-lines. Although luminal and basal cell-lines show different complements of individual features, the average constitutive levels of the 23 classes of lipids do not vary in the two groups (Additional file 1: Figure S1 and Additional file 2: Table S1).

### ATRA perturbs the lipidomic profiles of luminal and basal breast-cancer cell-lines

We compared the mass-spectrometry data obtained in all the cell-lines exposed to DMSO or ATRA (10^− 6^ M) for 48 h. The selection of the retinoid concentration and exposure time is based on pilot studies conducted in *SK-BR-3* cells. These studies demonstrate that maximal alterations in the lipidomic profiles are observed with 10^− 6^ M ATRA, while the 48-h exposure time precedes any significant effect on the number of cells [11].

**Fig. 3:**
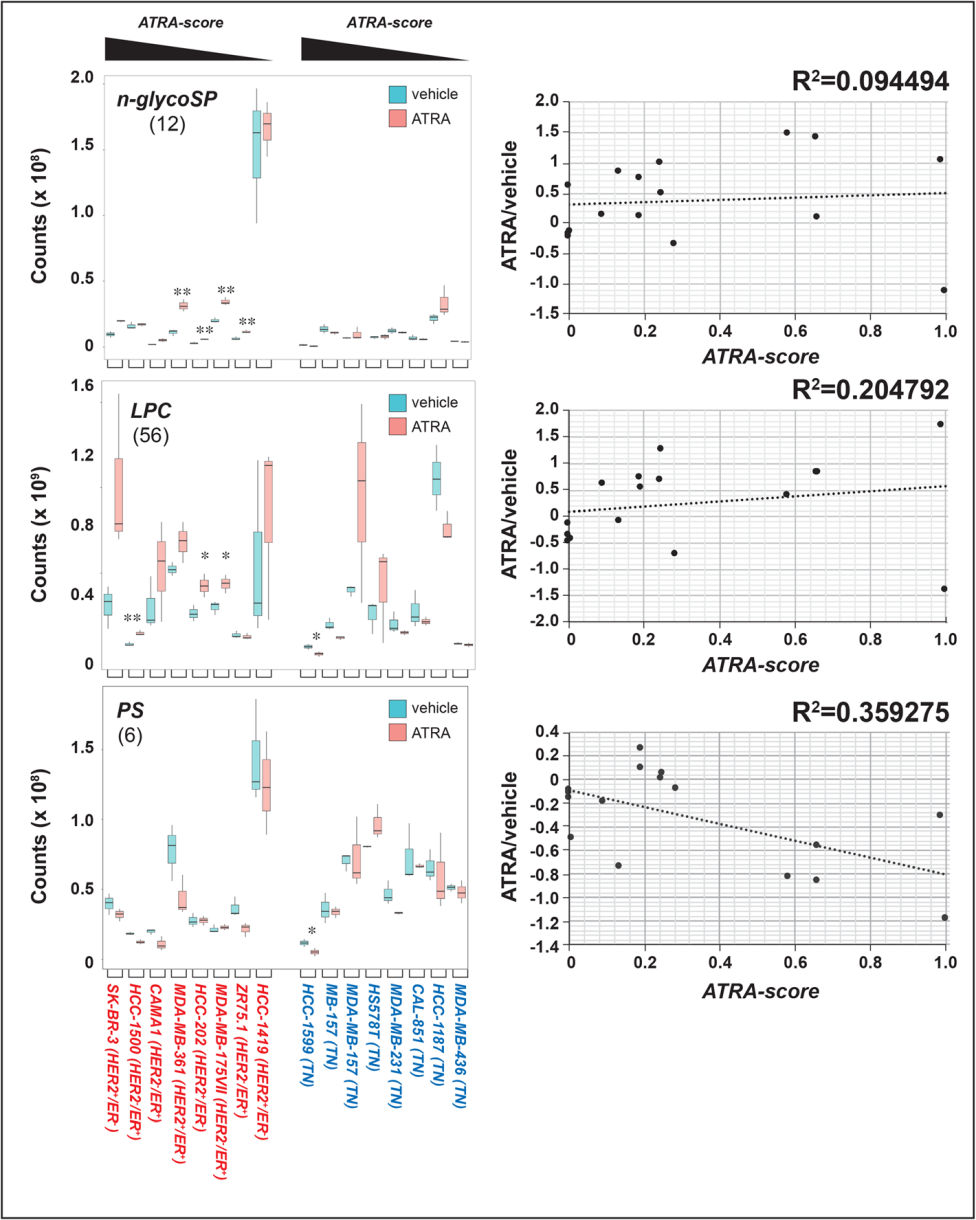
ATRA effects on the levels of N-glyco-sphingolipids, lysophoshatidylcholines and phosphatidylserines. Biological triplicates of the indicated breast cancer cells were treated with vehicle (DMSO) or ATRA (10^− 6^ M) for 48 h. The box plots show the median ± SD levels of neutral glyco-sphingosines (*N-Glyco-SP*), lysophoshatidylcholines *(LPC*) and phosphatidylserines (*PS*). The number of different molecules identified by mass-spectrometry is indicated in parenthesis. Luminal cell-lines are marked in red and basal cell-lines are marked in blue. The luminal and basal cell-lines are ordered according to decreasing sensitivity to the anti-proliferative effect of ATRA from left to right, as indicated (decreasing *ATRA-score*). *Significantly different (*p* < 0.05) from the corresponding vehicle treated control using the Student’s t-test. **Significantly different (*p* < 0.01) from the corresponding vehicle treated control using the Student’s t-test. The diagrams on the right indicate the correlations between the ATRA/DMSO ratio of the mean values calculated for the indicated lipid class in each cell-line and the corresponding *ATRA-score*

**Fig. 4:**
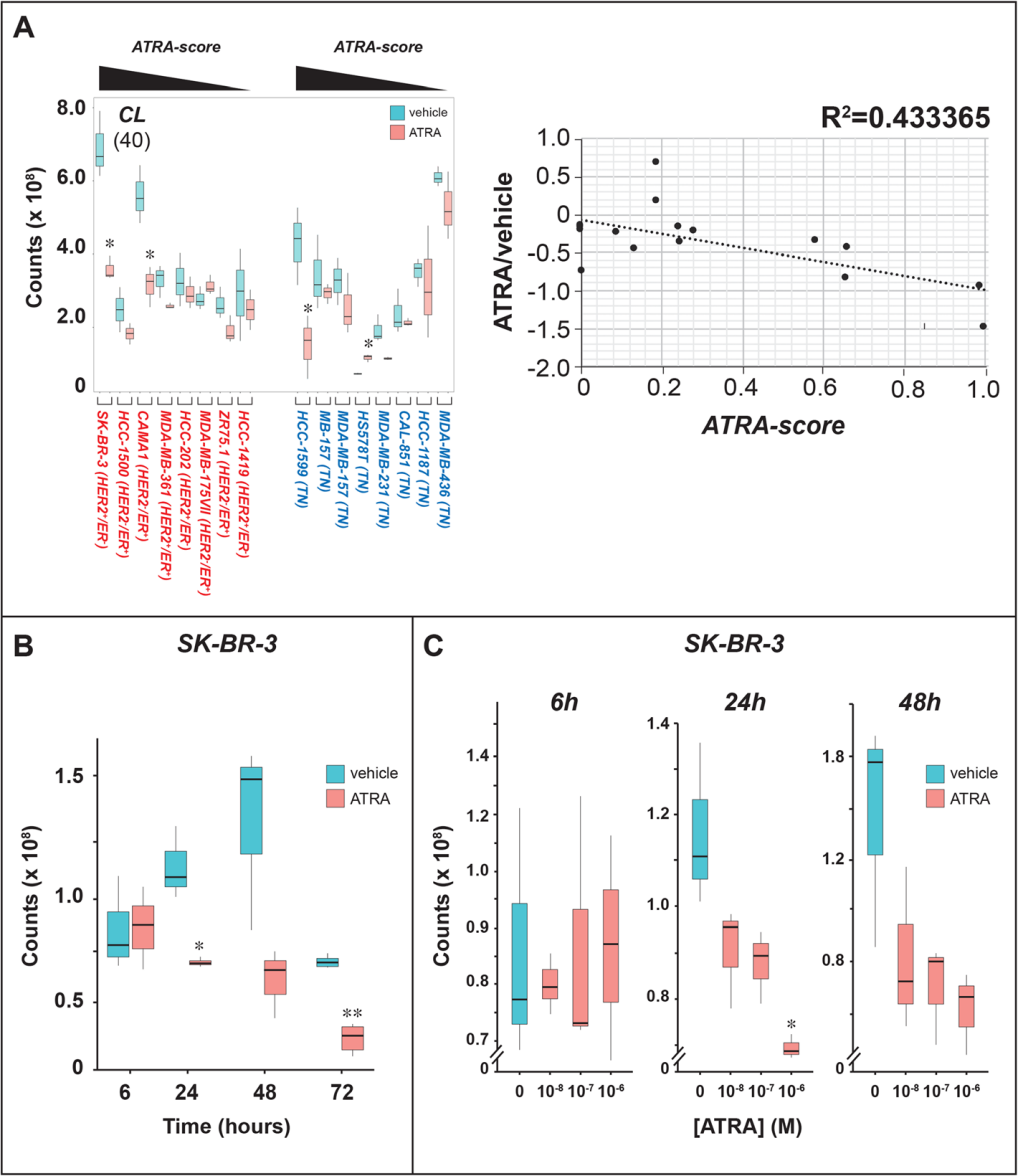
Effect of ATRA on the random motility of breast cancer cells. Biological triplicates of the indicated luminal (*MDA-MB-361*, *MDA-MB-175VII* and *HCC-1419*; marked in red) and basal (*MDA-MB-157*; marked in blue) cell lines. Cells were pre-treated with vehicle (DMSO) or ATRA. Each point is the Mean + SD of 40 cells. ***Significantly lower than the vehicle curve (*p* < 0.001 following two-way ANOVA Bonferroni post-test)

ATRA causes significant alterations of the lipidomic profiles in all the cell-lines considered (PCA, Additional file 1: Figure S2 and Additional file 2: Table S1). The retinoid determines the largest perturbations in cell-lines characterized by high/intermediate ATRA-sensitivity, regardless of the luminal or basal phenotype. Indeed, the effects triggered by ATRA in low-sensitivity/resistant cells are less evident, which is consistent with an association between the changes in the lipidomic profiles and the retinoid-dependent anti-proliferative effects (Additional file 1: Figure S2). We evaluated the effects exerted by ATRA on each of the 23 classes of lipids identified (Additional file 1: Figures S3-S5). For the majority of the classes, ATRA either triggers variable and cell-specific alterations in the total levels of the corresponding lipid components or causes no perturbation at all (Additional file 1: Figures S6-S8). The most interesting effects of ATRA are observed with *N-glycoSPs*, *LPCs*, *PSs* (Fig. 3) and *CLs* (Fig. 4)*.*

*N-glyco-SPs* are a group of cell-membrane sphingolipids and participate in biological processes such as cell adhesion and cell-cell interactions [35]. With the exception of retinoid-resistant *HCC-1419* cells, ATRA increases *N-glyco-SPs* levels in all luminal cell-lines (Fig. 3, left). *HCC-1187* is the only basal cell-line where a similar effect is observed. Hence, the ATRA-dependent increase of *N-glyco-SPs* is specific to luminal cells and it is not associated with the growth-inhibitory action exerted by ATRA in this breast cancer cell type. The point is illustrated by the low R^2^ correlation index calculated for the ATRA/DMSO ratio of the *N-glyco-SP* mean values determined in each cell-line and the corresponding *ATRA-Score* (Fig. 3, right).

*LPCs* result from phospholipase A2-dependent partial hydrolysis of phosphatidylcholines, which removes one of the fatty acid groups [36]. Similar to what is observed with *N-glycoSPs*, there is a trend towards a retinoid-dependent increase in the levels of *LPCs* in all luminal cell-lines, regardless of their sensitivity to ATRA (Fig. 3, left). The sole exception is represented by the *ZR75.1* cell-line, which shows no alteration in the amounts of *LPCs* following exposure to ATRA. The effects observed in basal cell-lines are variable and devoid of any association with the ATRA-dependent anti-proliferative action. Taken together the data suggest that the perturbations afforded by ATRA on *LPC* levels are not involved in the anti-proliferative action of the retinoid in either luminal or basal cell-lines. The conclusion is supported by the relatively low R^2^ correlation index between the ATRA/DMSO ratio of the *LPC* mean values and the *ATRA-score* (Fig. 3, right). The *LPCs* up-regulation observed in luminal cells may be related to ATRA metabolism and bio-disposition. In fact, *LPCs* are by-products of the transacylation reaction catalyzed by lecithin retinol acyl transferase (*LRAT*) which results in the conversion of all-trans retinol into all-trans retinyl ester, a storage form of Vitamin A [37].

*PSs* are cell membrane glycerophospholipids differing in their fatty acid composition and they are involved in cell-cycle signaling and apoptosis [38, 39]. In the four luminal and highly retinoid-sensitive *SK-BR-3*, *HCC-1500*, *CAMA1* and *MDA-MB-361* cell-lines, ATRA causes a reproducible reduction in the total amounts of the *PSs* identified (Fig. 3, left). With the exception of *ZR75.1*, a similar effect is not observed in the other luminal cell-lines characterized by intermediate/low sensitivity to the growth-inhibitory action of ATRA. *HCC-1599* is the only basal cell-line showing an ATRA-dependent decrease in *PSs*. The data obtained result in a relatively elevated R^2^ coefficient of correlation between the ATRA/DMSO ratio of the *PS* mean values determined in each cell-line and the corresponding *ATRA-Score* (Fig. 3, right). Taken together, our results suggest that the retinoid-dependent alterations in the levels of *PSs* play a role in the anti-proliferative effect exerted by ATRA predominantly in the context of luminal breast cancer cells. However, this hypothesis needs to be supported by further functional studies.

### ATRA reduces the motility of cell lines which respond to ATRA with an increase in N-glyco-SPs

Given the potential significance of *N-glyco-SPs* in cell adhesion and cell-cell interactions, the retinoid-dependent up-regulation of this particular subset of lipids in luminal breast cancer cells may have implications for the anti-metastatic and anti-motility effect exerted by ATRA in this type of mammary tumor [27]. To support this idea, we performed studies on the effects exerted by ATRA on the random-motility of four cell lines. To this purpose, we selected the luminal *MDA-MB-361* and *MDA-MB-175VII* cell lines, which are characterized by *N-glyco-SP* up-regulation upon ATRA exposure, as well as the luminal *HCC-1419* and the basal *MDA-MB-157* cells, which do not show a retinoid-dependent induction of the same type of lipids. Cells were pretreated with ATRA (10^− 6^ M) or vehicle (DMSO) for 16 h and subjected to a random-motility assay for another 24 h (Fig. 4). ATRA causes a significant reduction in the motility of *MDA-MB-361* and *MDA-MB-175VII* cells, while the retinoid does not alter the motility of *HCC-1419* and *MDA-MB-157* cells. In addition, we previously demonstrated [27] that ATRA reduces the directional motility of *SK-BR-3,* a luminal cell line whose *N-glyco-SP* levels are up-regulated by ATRA, although the up-regultaion does not reach statistical significance in our experimental conditions (Fig. 3).

Taken together, the cell-motility data support the idea that up-regulation *N-glyco-SPs* contribute to the anti-motility and anti-metastatic action of ATRA in luminal breast cancer cells.

### ATRA causes a specific down-regulation of CLs in sensitive breast-cancer cell-lines

**Fig. 5:**
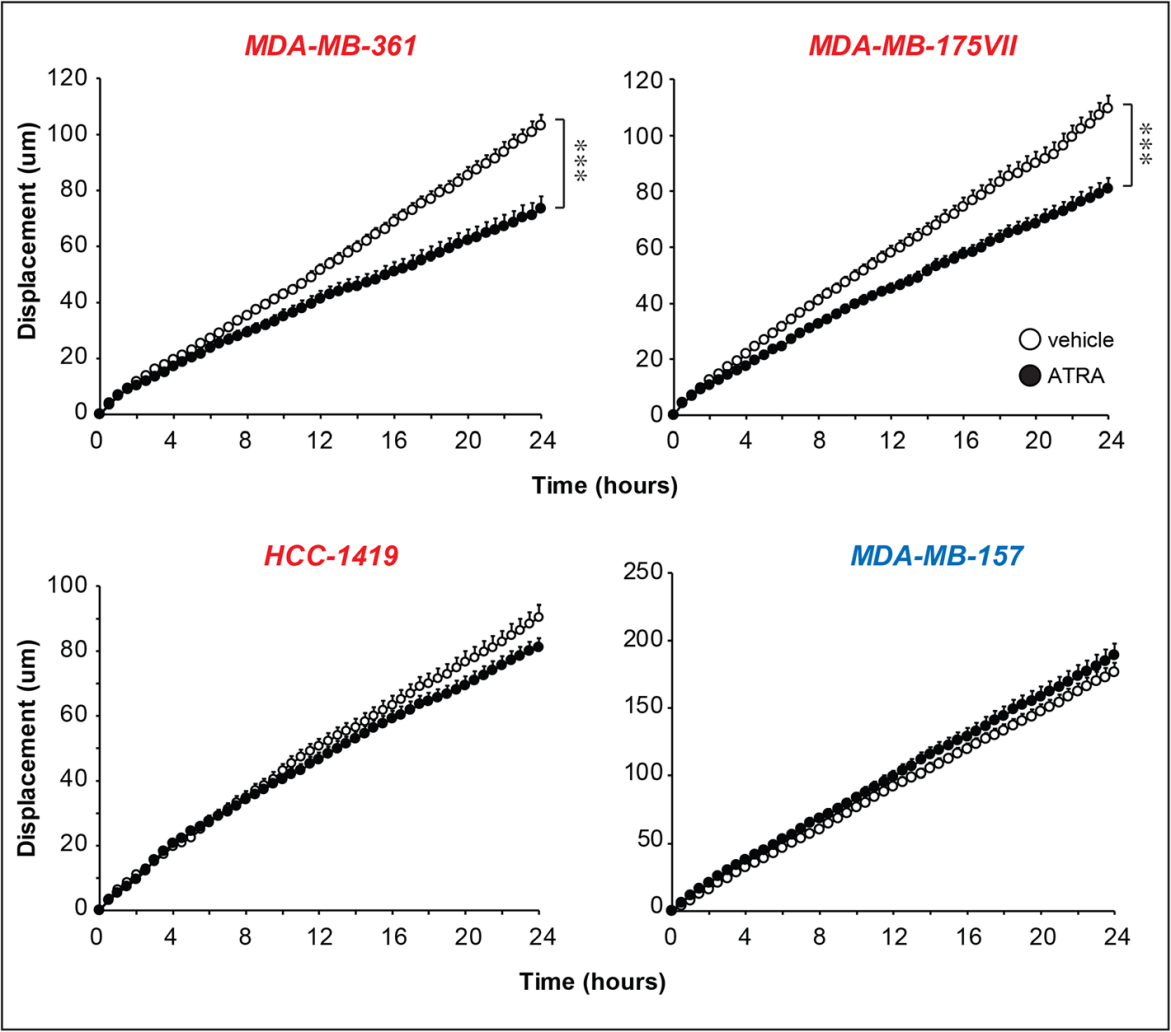
ATRA effects on the levels of cardiolipins. **a** Biological triplicates of the indicated breast cancer cells were treated with vehicle (DMSO) or ATRA (10^− 6^ M) for 48 h. Left: The box plots show the median ± SD levels of cardiolipins (*CLs*). The number of different *CL* molecules identified by mass-spectrometry is indicated in parenthesis. Luminal cell-lines are marked in red and basal cell-lines are marked in blue. The luminal and basal cell-lines are ordered according to decreasing sensitivity to the anti-proliferative effect of ATRA from left to right, as indicated (decreasing *ATRA-score*). Right: The diagram indicates the correlations between the ATRA/DMSO ratio of the mean values calculated for CLs in each cell-line and the corresponding *ATRA-score*. **b** Biological triplicates of SK-BR-3 cells were treated with vehicle (DMSO) or ATRA (10^− 6^ M) for the indicated amounts of time. The box plot shows the median ± SD levels of cardiolipins (*CLs*). **c** Biological triplicates of *SK-BR-3* cells were treated with vehicle (DMSO) or the indicated concentrations of ATRA for 48 h. The box plot shows the median ± SD levels of cardiolipins (*CLs*). *Significantly different (*p* < 0.05) from the corresponding vehicle treated control using the Student’s t-test. **Significantly different (*p* < 0.01) from the corresponding vehicle treated control using the Student’s t-test

The most interesting pattern of lipid perturbations afforded by ATRA involves *CLs*, a set of glycerophospholipids consisting of two phosphatidic acid moieties connected to a glycerol backbone (Additional file 1: Figure S9). Given the presence of 4 distinct alkyl chains, the potential complexity of *CL* individual species is enormous. However, in most animal tissues, *CLs* contain 18-carbon fatty alkyl chains each characterized by 2 unsaturated bonds [40]. Our lipidomic analysis identifies 40 *CL* species whose constitutive levels vary in each cell-line and are differentially modulated by ATRA (Additional file 2: Table S1). Across our panel of cell-lines, *CLs* show the highest levels of correlation with ATRA anti-proliferative effects. In fact, ATRA reduces the overall amounts of *CLs* in the luminal and highly sensitive, *SK-BR-3*, *HCC-1500*, *CAMA1* and *MDA-MB-361* cells. A similar ATRA-dependent reduction is observed in basal *HCC-1599* and *MDA-MB-157* cells (Fig. 5a, left). In addition, the ATRA/DMSO ratio of the *CL* mean-values in each cell-line is inversely correlated with the corresponding *ATRA-scores* and shows a high R^2^ index (Fig. 5a, right). The observed association supports the idea that the reduction in the overall amounts of *CLs* contributes to the anti-proliferative action of ATRA in both luminal and basal cell-lines. We evaluated the time and concentration dependence of the *CLs* decrease induced by ATRA in luminal and retinoid-sensitive *SK-BR-3* cells (Additional file 3: Table S2). In this cellular context, the effect of ATRA is relatively late and long-lasting, as a significant reduction in *CL* levels is evident only at 24 h and it is maintained until 72 h (Fig. 5b). In addition, *CLs* down-regulation is concentration dependent, as indicated by the results obtained following exposure of *SK-BR-3* cells to increasing amounts of ATRA (Fig. 5c).

### The ATRA-dependent decrease in CL levels is accompanied by down-regulation of genes involved in mitochondrial oxidative phosphorylation

We focused our attention on the specific down-regulation of *CLs* afforded by ATRA in sensitive cell-lines. *CLs* are part of the composite glycerophospholipid metabolic pathway (https://www.genome.jp/kegg/pathway.html). To obtain insights into the mechanism underlying the ATRA-dependent down-regulation of *CLs*, we evaluated the RNA-sequencing data obtained in all the 16 cell-lines exposed to ATRA (10^− 6^ M) or vehicle for 24 h (M. Bolis et al., Array Express ref. No: E-MTAB-8408). Using these data, we determined the effects of ATRA on the expression of the 77 genes involved in the glycerophospholipid metabolic pathway (Additional file 1: Figure S10).

**Fig. 6:**
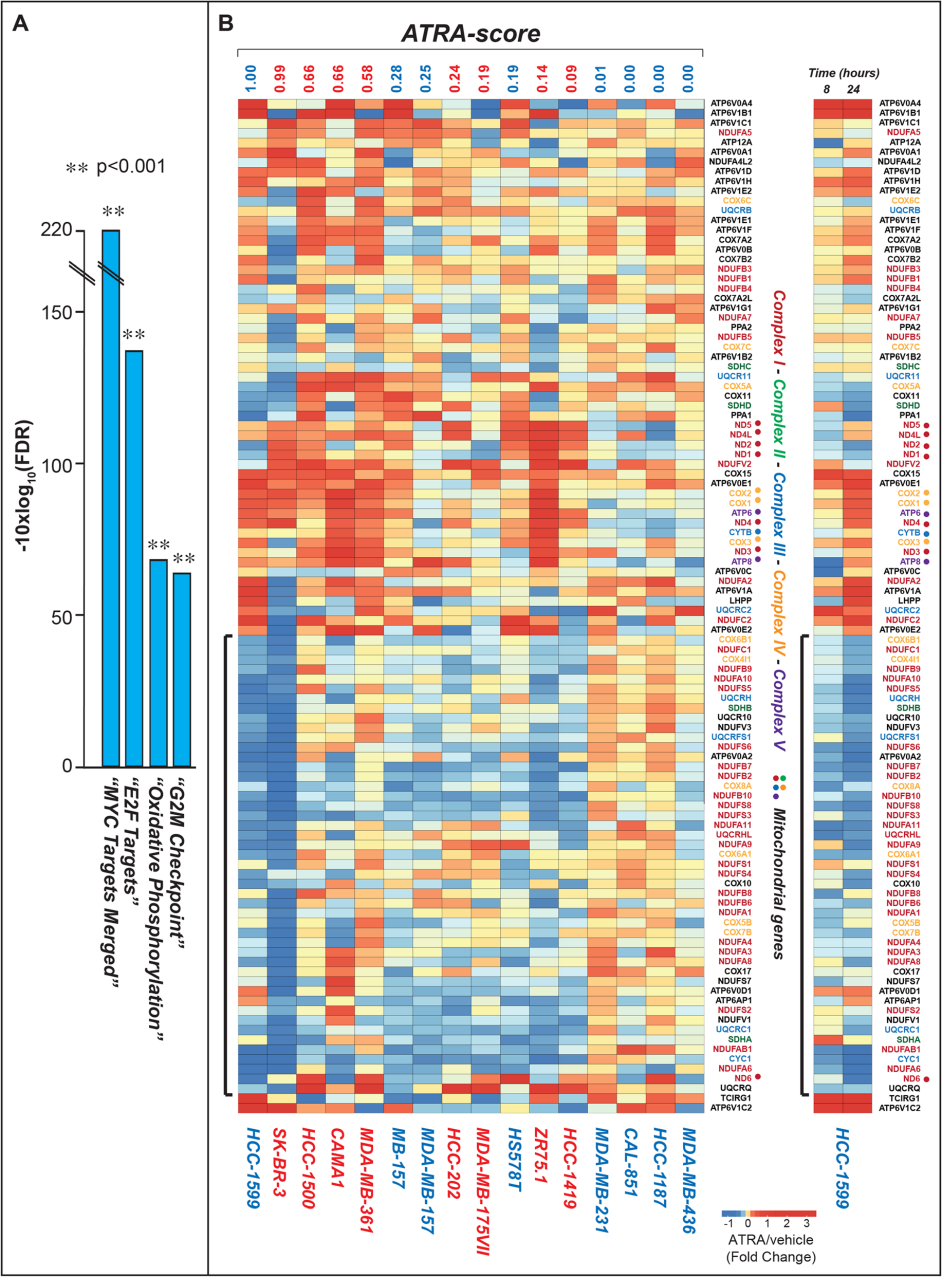
Effects of ATRA on the expression of genes involved in the mitochondrial oxidative phosphorylation. Biological triplicates of the indicated breast cancer cells treated with vehicle (DMSO) or ATRA (10^− 6^ M) for 24 h. Total RNA was extracted and subjected to RNA-seq analysis. **a** The figure shows the 4 top Hallmark pathways significantly enriched for genes down-regulated by ATRA in sensitive luminal and basal cell-lines ranked according to the enrichment False Discovery Rate (FDR) *p*-value. **b** Left: The heat-map shows the expression profiles of the genes belonging to the *“Oxidative-Phosphorylation”* Hallmark pathway. The results are expressed in log_2_ values of the ATRA/DMSO ratio as indicated. Right: The heat-map shows the expression profiles of the *“Oxidative-Phosphorylation”* observed in *HCC-1599* cells exposed to ATRA (10^− 6^ M) for 8 and 24 h. The results are expressed in log2 values of the ATRA/DMSO ratio as indicated. The genes coding for members of the mitochondrial complexes I, II, III, IV and V are marked in different colors. When the genes encode mitochondrial proteins which do not belong to any complex, they are marked in black. The coding genes transcribed from mitochondrial DNA are further indicated by a colored point. The genes down-regulated by ATRA in retinoid sensitive cell-lines are indicated by a black square bracket on the left

Although specific genes are significantly up- or down-regulated by ATRA in single cell-lines (for instance see the expression pattern of MBOAT1, Additional file 1: Figure S10), there is no significant correlation (low R^2^ values) between the ATRA/DMSO expression ratio of any of these genes and the *ATRA-score* values. The results support the idea that the ATRA-dependent *CLs* decrease observed in retinoid sensitive cell-lines cannot be explained by up-regulation of specific genes controlling the catabolism of *CLs* or down-regulation of genes involved in their biosynthesis. *CLs* are predominantly located in the inner membrane of mitochondria, where they are involved in oxidative phosphorylation [41]. This suggests that the anti-proliferative effect exerted by ATRA in sensitive cell-lines may be accompanied by perturbations in mitochondrial homeostasis. Pathway enrichment analysis of the RNA-sequencing data supports this idea. In fact, “*Oxidative Phosphorylation*” lays in third position among the 4 top Hallmark gene-sets enriched for genes down-regulated by ATRA in sensitive luminal and basal cell-lines (Fig. 6a). The other 3 top gene-sets collectively down-regulated by ATRA are “*Myc Targets*”, “*E2F Targets*” and “*G2M Checkpoint*”. The down-regulation of these gene-sets, which control cell-cycle and proliferation, is likely to be associated with the growth-inhibitory action exerted by ATRA in sensitive cell-lines.

The “*Oxidative Phosphorylation*” gene-set consists of 135 genes coding for mitochondrial proteins [42]. The vast majority of mitochondrial proteins are encoded by nuclear genes. Oxidative Phosphorylation is responsible for the production of ATP from NADH *via* the activity of 5 large protein complexes (mitochondrial complexes I-V) situated in the inner mitochondrial membrane. ATRA down-regulates approximately half of the “Oxidative Phosphorylation” genes in the luminal and basal cell-lines characterized by high/intermediate sensitivity to the retinoid (see black square brackets in Fig. 6b). In these cell-lines, the level of down-regulation correlates with retinoid-sensitivity. Indeed, the highest downregulation is observed in basal *HCC-1599* and luminal *SK-BR-3* cells, which are characterized by the 2 top *ATRA-scores*. With the exception of *ND6*, all the genes down-regulated by ATRA in sensitive cells are of nuclear origin. Down-regulation of the “Oxidative Phosphorylation” gene-network by ATRA is a relatively early event, as indicated by the RNA-sequencing data obtained in *HCC-1599* cells exposed to ATRA (10^− 6^ M) for 8 h (Fig. 6b). In fact, a significant decrease in the levels of most of the down-regulated genes is already evident at this time point. This is consistent with the idea that ATRA causes a rapid transcriptional repression of various nuclear genes coding for mitochondrial proteins.

### ATRA-dependent reduction of CLs is associated with a decrease in the number and activity of mitochondria

Given the observed down-regulation of *CLs* and the expression of multiple genes involved in oxidative phosphorylation, we evaluated the action of ATRA on the amounts and function of mitochondria in selected luminal cell-lines characterized by sensitivity and resistance to the anti-proliferative effects of the retinoid. We focused our attention on two couples of homogeneous luminal and HER2^+^ cell-lines (*SK-BR-3*/*HCC-1419* and *MDA-MB-361*/*HCC-202*). Indeed, PCA demonstrates that the constitutive lipidomic profiles of the ATRA-sensitive *SK-BR-3* and the ATRA-insensitive *HCC-1419* cell-lines are very similar (Fig. 2b). Similar constitutive lipidomic profiles are also observed in ATRA-sensitive *MDA-MB-361* cells and the much less responsive *HCC-202* counterparts.

**Fig. 7:**
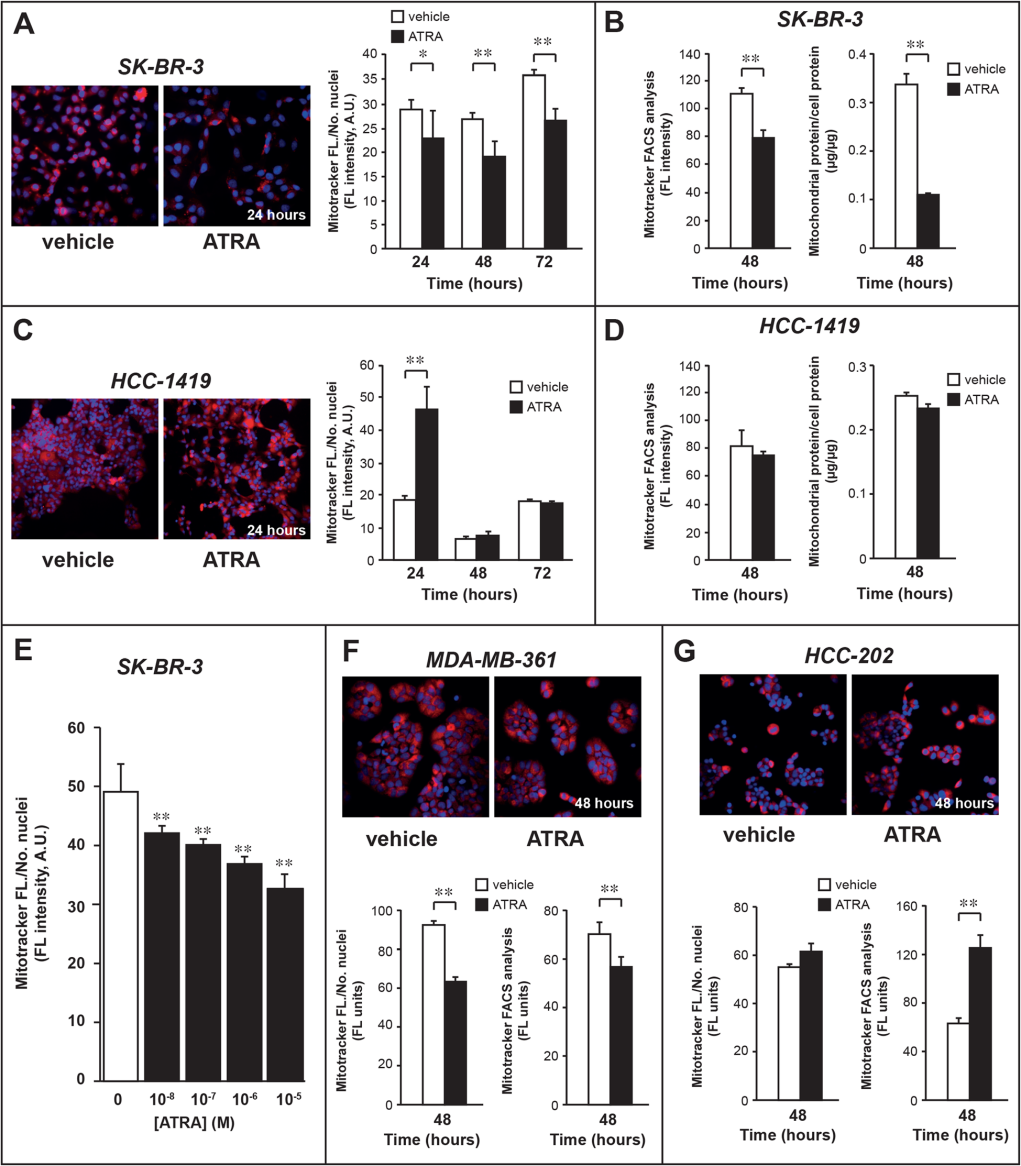
Effects of ATRA on the number of mitochondria. **a** and **c** Biological triplicates of retinoid-sensitive *SK-BR-3* and retinoid-resistant *HCC-1419* cells were treated with vehicle (DMSO) or ATRA (10^− 6^ M) for the indicated amount of time. The left pictures show representative fluorescence images of the indicated *SK-BR-3* and *HCC-1419* cells treated for 24 h before staining with Hoechst for the cell nuclei (blue fluorescence) and Mitotracker to highlight the mitochondria (red fluorescence). The column diagrams on the right indicate the time-course of the effect exerted by ATRA on the number of mitochondria. The results were obtained from the quantitative image-analysis of Mitotracker fluorescence in at least 4 fields/experimental triplicate. **b** and **d** The left diagrams indicate the effect exerted by ATRA on the number of mitochondria in *SK-BR-3* (**b**) and *HCC-1419* (**d**) cells, as assessed by FACS analysis of Mitotracker fluorescence (Mean ± SD, *N* = 3), following 48 h of treatment. The right diagrams illustrate the total amounts of proteins determined in mitochondria isolated from *SK-BR-3* (**b**) and *HCC-1419* (**d**) cells. Mitochondria were isolated from the same number of cells and the amount of mitochondrial proteins was determined. The results are expressed as the ratio of mitochondrial protein over the total amount of cellular proteins. **e** Three independent cultures of *SK-BR-3* cells per experimental point were treated with the indicated concentrations of ATRA for 48 h. At the end of the treatment, cells were stained with Hoechst for the cell nuclei (blue fluorescence) and Mitotracker to highlight the mitochondria (red fluorescence) as in (**a**) and (**c**). The column diagram shows the effect exerted by ATRA on the number of mitochondria. The results were obtained from the quantitative image-analysis of Mitotracker fluorescence in at least 4 fields/experimental triplicate. **f** and **g** Biological triplicates of retinoid-sensitive *MD-MB-361* and the retinoid-resistant *HCC-202* cells were treated with vehicle (DMSO) or ATRA (10^− 6^ M) for 48 h. The upper pictures show representative fluorescence images of the indicated *MD-MB-361* and *HCC-202* cells treated for 24 h before staining with Hoechst and Mitotracker. The lower left diagrams show the effects exerted by ATRA on the number of mitochondria, as in (**a**), (**b**) and (**e**). The results were obtained from the quantitative image-analysis of Mitotracker fluorescence in at least 4 fields/experimental triplicate. The lower right diagrams indicate the effect exerted by ATRA on the number of mitochondria in *MD-MB-361* (**f**) and *HCC-202* (**g**) cells, as assessed by FACS analysis of Mitotracker fluorescence (Mean ± SD, *N* = 3), following 48 h of treatment. Each value is expressed as the Mean + SD (N = 3). *Significantly different (*p* < 0.05, Student’s t-test); **Significantly different (*p* < 0.01, Student’s t-test)

To determine the amounts of mitochondria, *SK-BR-3* and *HCC-1419* cells were exposed to ATRA (10^− 6^ M) for 24, 48 and 72 h, stained with Mitotracker and subjected to quantitative fluorescence microscopy (Fig. 7a and Fig. 7c). In *SK-BR-3* cells*,* ATRA causes a significant reduction in Mitotracker-associated fluorescence, which is already evident at 24 h and it is maintained at 48 and 72 h (Fig. 7a). The results obtained at 48 h are confirmed by FACS analysis of the Mitotracker-stained cells (Fig. 7b, left). These data are consistent with an ATRA-triggered decrease in the amounts of mitochondria. The phenomenon is validated by measurement of the total amounts of mitochondrial proteins (Fig. 7b, right). The decrease in the amounts of mitochondria caused by ATRA in *SK-BR-3* cells is dose-dependent and the phenomenon is already evident at 10^− 8^ M ATRA (Fig. 7e). The reduction in the number of mitochondria is likely to be one of the mechanisms at the basis of the anti-proliferative action exerted by ATRA, as indicated by the results obtained in *HCC-1419* cells. In fact, exposure of this ATRA-insensitive cell-line to the retinoid causes an early and paradoxical increase in Mitotracker-associated fluorescence, which is observed at 24 h and reverts to baseline by 48 h (Fig. 7c). In addition, FACS analysis shows no difference between *HCC-1419* cells exposed to vehicle or ATRA for 48 h (Fig. 7d, left) and the amounts of total mitochondrial proteins are not altered by the retinoid (Fig. 7d, right). The results of the *SK-BR-3/HCC-1419* cell pair are consistent with what is observed in the *MDA-MB-361*/*HCC-202* counterpart exposed to 10^− 6^ M ATRA for 48 h. In fact, the data obtained with the use of quantitative immuno-histochemical (Fig. 7f/g, lower leftmost diagrams) and FACS (Fig. 7f/g, lower rightmost diagrams) analyses demonstrate a reduction in the amounts of mitochondria only in the sensitive *MDA-MB-361*.

**Fig. 8:**
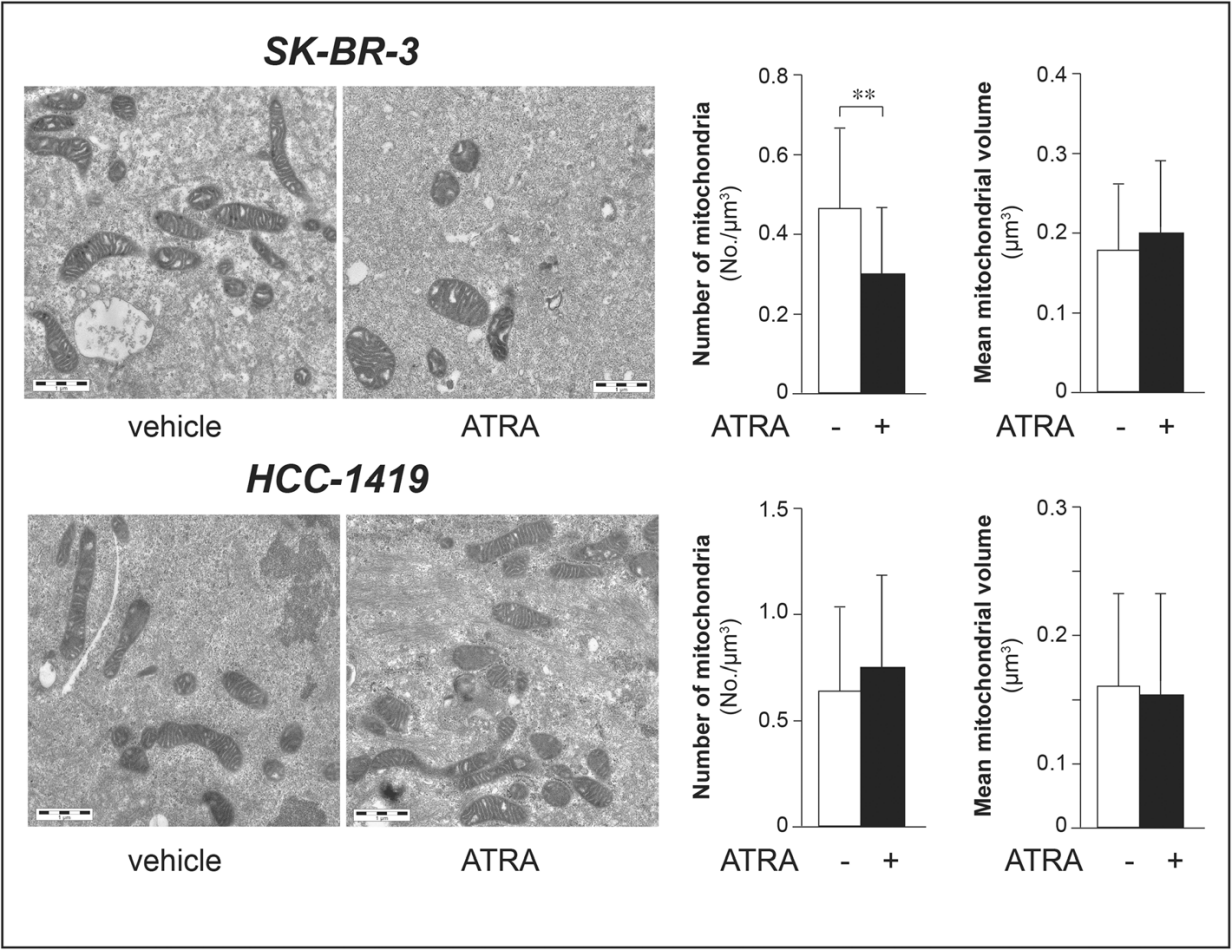
Electron-microscopy of mitochondria in SKBR-3 and HCC-1419 cells. The two *SK-BR-3* and *HCC-1419* cell-lines were treated with vehicle (DMSO) or ATRA (10^− 6^ M) for 48 h. The photographs show electron-microscopy representative images from three replicate cultures of retinoid-sensitive *SK-BR-3* and retinoid-resistant *HCC-1419* cells. The images illustrate the ultrastructure of mitochondria which show no alterations in the outer membranes, cristae and matrix in either DMSO treated or ATRA treated cells. The left column bargraphs indicate the number of mitochondria measured in *SK-BR-3* (upper) and *HCC-1419* (lower) cells calculated as the numerical density of mitochondria (N_V_, n/μm^3^) estimated from the morphometrical analysis performed on 30 digitized electron microscope fields/experimental group. The right column bargraphs indicate the volume of mitochondria measured in *SK-BR-3* (upper) and *HCC-1419* (lower) cells on the same number of electron microscope fields as above. Each value is the Mean ± SD of 30 cells. **Significantly lower than the vehicle value (*p* < 0.01 following Student’s t-test analysis)

To validate and extend the data obtained on mitochondria, we performed quantitative morphological studies on these organelles following isolation from the retinoid-sensitive *SK-BR-3* and the retinoid-resistant *HCC-1419* cell lines exposed to vehicle or ATRA (10^− 6^ M) for 48 h (Fig. 8). The transmission electron microscopy results obtained confirm that ATRA causes a reduction in the number of mitochondria in *SK-BR-3* cells, as indicated by the significant decrease in the numerical density of these organelles. Conversely, the retinoid does not alter the number of mitochondria in *HCC-1419* cells (Fig. 8, left column graphs). In addition, ATRA exerts no significant effect on the size of mitochondria in either cell line (Fig. 8, right column graphs). Finally, the ATRA-dependent reduction in the amounts of mitochondria observed in *SK-BR-3* cells is unlikely to be due to an increase in the degradation of these organelles by mitophagy following retinoid-dependent cell damage or stress [43–45]. In fact, the electron microscopy studies performed in *SK-BR-3* and *HCC-1419* cells demonstrate the presence of intact and morphologically fit mitochondria in both cell types regardless of ATRA treatment (Fig. 8, electron micrographs). By converse, down-regulation of numerous nuclear genes involved in oxidative phosphorylation (Fig. 6) suggests that ATRA reduces the assembly of mitochondria.

**Fig. 9:**
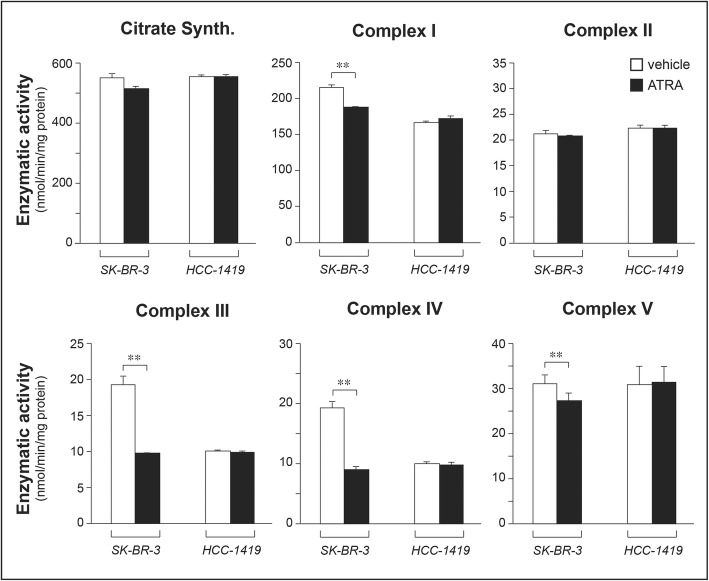
Effects of ATRA on mitochondrial COMPLEX I-V enzymatic activity. Biological triplicates of the retinoid-sensitive *SK-BR-3* and retinoid-resistant *HCC-1419* cells were treated with vehicle (DMSO) or ATRA (10^− 6^ M) for 48 h. At the end of the treatment mitochondria were isolated and subjected to the measurement of Complexes I-V and citrate synthetase (Citrate Synth.) enzymatic activities. The same amounts of mitochondrial proteins were used for the determination of the various enzymatic activities, as indicated by the same amount of citrate synthase measured in each experimental group. The enzymatic activity values are expressed as nmol/min per mg protein of substrate metabolized. All the values of COMPLEX I-V are normalized for the content of citrate synthetase activity (Mean + SD, N = 3). *Significantly different (*p* < 0.05, Student’s t-test); **Significantly different (*p* < 0.01, Student’s t-test)

To evaluate whether ATRA exerts significant effects not only on the amounts, but also on the functional activity of mitochondria, we isolated these organelles from *SK-BR-3* and *HCC-1419* cells treated with vehicle or ATRA (10^− 6^ M) for 48 h. We measured the enzymatic activity of respiratory complexes I-V following normalization for the levels of citrate synthase activity (Fig. 9). In sensitive *SK-BR-3* cells, ATRA decreases the enzymatic activity of mitochondrial complexes I, III, IV and V. Similar effects are not observed in ATRA-insensitive *HCC-1419* cells. Interestingly, treatment of *SK-BR-3* cells with ATRA reduces the microviscosity of mitochondrial membranes (Additional file 1: Figure S11), a parameter controlled by *CLs*, which are known membrane rigidifying agents [46]. The ATRA-dependent decrease in microviscosity starts to be observed at 24 h and it is maintained at 48 h. The time course of the reduction in microviscosity and the decrease in *CL* levels is similar, suggesting that the two phenomena are associated. The decrease in microviscosity indicates that ATRA causes a relative deficit of mitochondrial *CL* levels which is likely to be at the basis of the retinoid-dependent reduction in the activity of complexes I, III, IV and V.

### The down-regulation of CLs and the decrease in mitochondrial membrane microviscosity by ATRA are dependent on RARα expression

The involvement of *CLs* down-regulation and decreased assembly/function of mitochondria in the sensitivity of breast-cancer cells to ATRA is supported by the data obtained in RARα over-expressing and RARα knock-down cells [11].

**Fig. 10:**
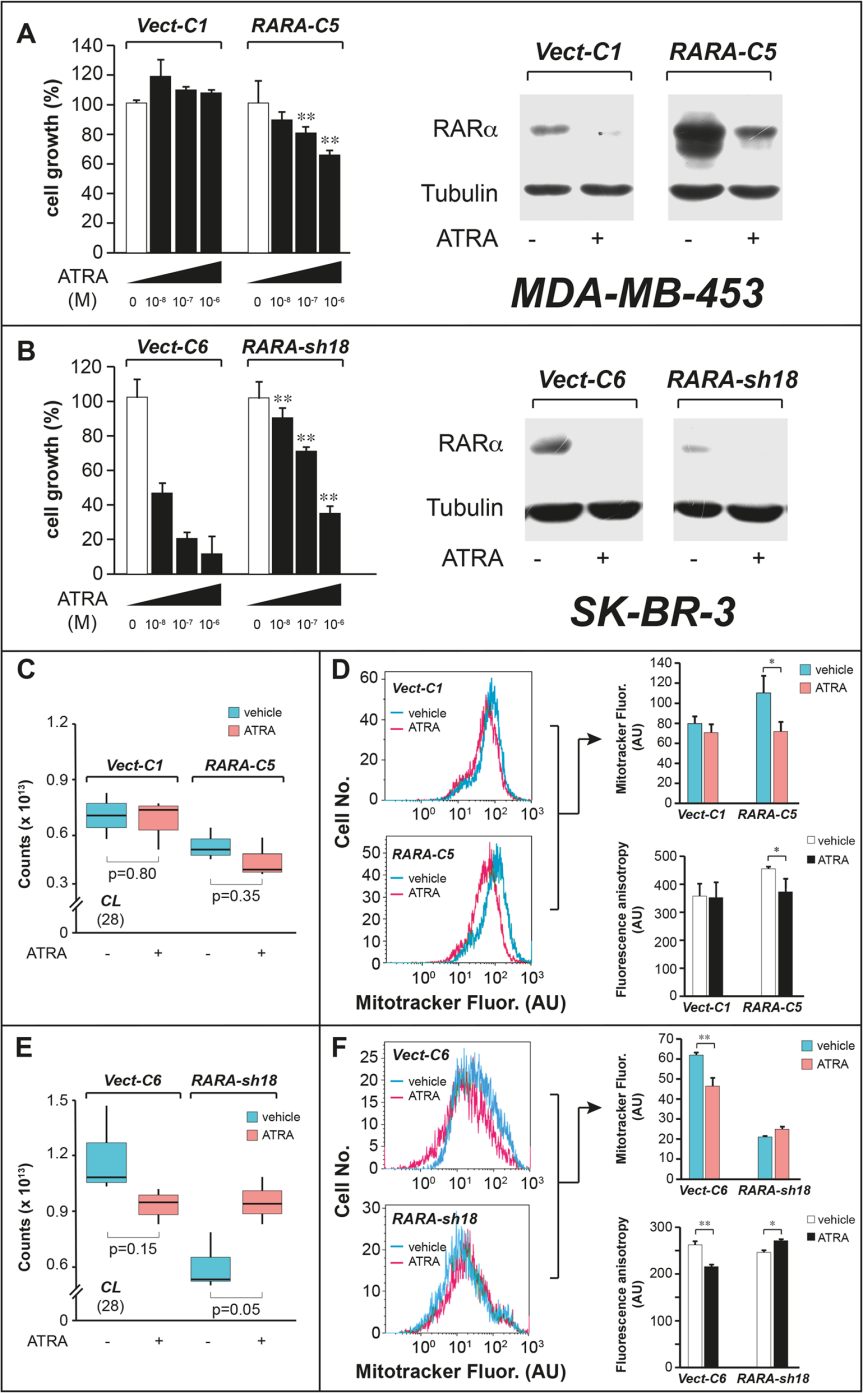
Effects of ATRA on the growth, CL levels, the amounts of mitochondria and the mitochondrial membrane microviscosity in RARα over expressing MDA-MB-453 and RARα knock-down SK-BR-3 cells. *RARA-C5* are *MDA-MB-453* cells stably transfected with a plasmid construct containing the RARα cDNA, while *Vect-C1* are control *MDA-MB-453* cells stably transfected with the void vector. *RARA-sh18* are *SK-BR-3* cells stably infected with a retrovirus allowing the expression of a shRNA targeting RARα, while *Vect-C6* are control *SK-BR-3* cells stably infected with the void retroviral vector. **a** and **b** Left: Triplicate cultures of the indicated cells were treated with vehicle and the indicated concentrations of ATRA for 6 days. At the end of the treatment cell growth was determined with the use of a sulforhodamine assay. Right: The amounts of RARα protein were determined by Western Blot analysis in the indicated cells following treatment with vehicle (DMSO or ATRA (10^− 6^ M) for 24 h. Tubulin was used as a loading control. **c** and **e** Biological triplicates of the indicated breast-cancer cells were treated with vehicle (DMSO) or ATRA (10^− 6^ M) for 48 h. The box plots show the median ± SD levels of cardiolipins (*CLs*). The number of different *CL* molecules identified by mass-spectrometry is indicated in parenthesis. **d** and **f** Biological triplicates of the indicated breast-cancer cells were treated with vehicle (DMSO) or ATRA (10^− 6^ M) for 48 h. At the end of the treatment, cells were stained with Mitotracker, fixed in 2% formalin and subjected to FACS analysis. The colored left diagram show representative FACS diagrams, while the colored right bar graphs show the quantitative results obtained in biological triplicates. As for the results presented in the black and white column bargraphs of panels **d** and **f**, three replicate cultures of the indicated cells were treated with vehicle (DMSO) or ATRA (10^− 6^ M). In particular, *Vect-C1* and *RARA-C5* cells were treated for 5 days, while *Vect-C6* and *RARA-sh18* cells were treated for 2 days. At the end of the treatment, mitochondria were isolated and incubated with 1,6-diphenyl-1,3,5-hexatriene to assess the microviscosity of mitochondrial membranes. The values are expressed as the Mean ± SD of the membrane microviscosity values, following measurement of fluorescence anisotropy (N = 3). Panels (**a**-**f**): **Significantly different relative the vehicle treated controls (*p* < 0.01, Student’s t-test). *Significantly different relative the vehicle treated controls (*p* < 0.05, Student’s t-test)

In a first set of studies, we took *MDA-MB-453* cells stably transfected with a plasmid construct allowing the expression of RARα (*RARA-C5*) or the corresponding void vector (*Vect-C1*) [11]. As expected, *RARA-C5* cells are characterized by higher RARα expression levels than the *Vect-C1* counterparts and the nuclear retinoid receptor is functionally active as indicated by its ATRA-dependent degradation (Fig. 10a, right), a phenomenon associated with transcriptional activation [47, 48]. In line with what is observed in parental *MDA-MB-453* cells [11], which are characterized by low RARα levels and ATRA-resistance, *Vect-C1* cells do not respond to the retinoid (Fig. 10a, left). In contrast, RARα over-expression renders *RARA-C5* cells partially responsive to ATRA growth-inhibitory action [11]. The lipidomic data demonstrate that exposure of the two cell-lines to ATRA (10^− 6^ M) for 48 h does not alter the overall levels of *CLs* in *Vect-C1* cells, while it causes a reproducible decrease in *RARA-C5* cells (Fig. 10c and Additional file 4: Table S3). In the same experimental conditions, ATRA reduces the number of mitochondria in Mitotracker stained *RARA-C5*, but not in *Vect-C1* cells (Fig. 10d). In addition, ATRA reduces the microviscosity of mitochondrial membranes only in retinoid-responsive *RARA-C5* cells (Fig. 10d, black and white column graph).

We conducted similar types of studies in *SK-BR-3* cells stably infected with a retroviral construct containing a RARα-targeting shRNA (*RARA-sh18*) and the corresponding void vector (*Vect-C6*) [11]. As expected, *RARA-sh18* are characterized by lower levels of functionally active RARα than the corresponding *Vect-C6* control cell-line (Fig. 10b, right). Consistent with the high *ATRA-score* of parental *SK-BR-3* cells (Fig. 1b) [11], the growth of *Vect-C6* cells is decreased by ATRA in a dose-dependent fashion (Fig. 10b, left). RARα knock-down reduces the sensitivity of *RARA-sh18* cells to ATRA anti-proliferative effects. Similar to parental *SK-BR-3* cells (Fig. 5a), exposure of *Vect-C6* to ATRA (10^− 6^ M) for 48 h decreases *CL* levels (Fig. 10e, Additional file 4: Table S3). A decrease in the total amounts of *CLs* is not observed in *RARA-sh18* cells, where the retinoid causes an opposite effect (Fig. 10e). In the same experimental conditions, ATRA reduces the number of mitochondria in *Vect-C6* control cells, but not in *RARA-sh18* cells (Fig. 10f). The reduction of mitochondria in *Vect-C6* is accompanied by a decrease in mitochondrial membrane microviscosity, while this parameter is slightly increased in *RARA-sh18* cells (Fig. 10f, black and white column graph).

All these results indicate that the ATRA-induced decrease in *CLs* and the associated reduction in mitochondrial membrane microviscosity are dependent on RARα, which is a crucial determinant of ATRA anti-tumor activity in breast cancer [11].

## Discussion

ATRA is a promising anti-tumor agent and it is the object of an approved window-of-opportunity clinical trial we are conducting in the context of the personalized treatment of ER^+^ breast cancer patients > 65 years of age undergoing neo-adjuvant treatment with aromatase inhibitors (E. Garattini, personal communication). The present report provides new insights into the mechanisms underlying the anti-tumor action of ATRA, a non-conventional therapeutic agent [11, 12, 26]. The high-throughput lipidomic approach used in our study demonstrates that basal and luminal cell-lines, growing under standard conditions, are characterized not only by distinct gene-expression profiles, but also by a different complement of lipids, which is likely to be related to the corresponding mesenchymal and epithelial phenotypes. ATRA exerts profound and variable effects on the lipid composition of luminal and basal cells, altering the levels of numerous lipid species, which may be the result of the well-known differentiating action of the retinoid [49]. In particular, ATRA causes a significant and selective increase of N-glycoSPs in retinoid-sensitive luminal cells. This effect is likely to contribute to the anti-motility effects of ATRA, which is consistent with the hypothesized role of this lipid class in the invasive and metastatic properties of cancer cells [35].

The most interesting effects of ATRA are observed on *CLs*, a class of lipids predominantly located in the inner mitochondrial membrane and playing an important role in oxidative phosphorylation [41]. ATRA reduces the overall amounts of *CLs* and this effect is correlated to the growth inhibitory activity of the retinoid. Down-regulation of *CLs* is accompanied by a significant reduction in the number and functional activity of mitochondria, two phenomena which are likely to contribute to the growth inhibitory action of ATRA in sensitive cell-lines. Our transcriptomic data indicate that ATRA-dependent down-regulation of *CLs* is consequent to a decrease in the assembly of mitochondria. In fact, ATRA does not exert direct effects on the expression of genes involved in the biosynthesis/catabolism of *CLs*, while the retinoid down-regulates numerous genes coding for mitochondrial proteins via direct or indirect transcriptional events mediated by RARα. In addition, the ATRA-dependent down-regulation of nuclear genes encoding mitochondrial proteins is a relatively early event, preceding the decrease in *CL* levels. Although *CLs* down-regulation is unlikely to be a determinant of the reduction in the amounts of mitochondria, the process may contribute to the functional deficiency of these organelles induced by ATRA in sensitive cells. In fact, the decrease in the enzymatic activity of mitochondrial complexes I, III IV and V observed in *SK-BR-3* cells may be explained by the fact that ATRA induces a relative deficit of *CLs* in the mitochondrion. This, in turn, reduces the microviscosity of mitochondrial membranes, a phenomenon which is potentially at the basis of the ATRA induced decrease in mitochondrial complexes activity [50–52].

The molecular mechanisms underlying the reduction in the number of mitochondria and the consequent decrease in the levels of *CLs* caused by ATRA must be further investigated. Nevertheless, we recently observed that exposure of retinoid-sensitive luminal and basal breast cancer cell lines to ATRA induces the expression of various endogenous retroviral RNAs, resulting in an unusual type of “*viral-mimicry*” [53] response (M. Terao and M. Bolis, unpublished results). This ATRA-dependent process of “*viral-mimicry*” causes the induction of the transcriptional factor, IRF1 (Interferon Responsive Factor 1), via RARα activation and it is associated with the up-regulation of numerous other interferon-responsive genes. Interestingly, viruses [54], IRF1 [55] and other interferon-responsive genes are involved in the control of mitochondrial homeostasis, regulating the number and viability of these intracellular organelles. Hence, we hypothesize that activation of “*viral-mimicry*” and the consequent cascade of transcriptional effects involving interferon-dependent genes are at the basis of the reduction in the number and functional activity of mitochondria observed in sensitive breast cancer cell lines exposed to ATRA.

The involvement of mitochondria in the anti-proliferative action exerted by ATRA on the neoplastic cell is of relevance for the development of innovative approaches in the treatment of breast-cancer. In fact, cancer cell mitochondria are novel therapeutic targets, given their structural and functional differences from the normal counterparts [56, 57]. The mitochondria of cancer and normal cell are also key regulators of the apoptotic pathway which is often defective in the former cellular context. Recently, mitochondria have been shown to represent important determinants for cancer stem-cell propagation raising further interest in the development of anti-tumor agents selectively targeting cancer cell mitochondria [58]. Among these new therapeutic agents, a novel arylurea-fatty-acid is of particular interest for our study. In fact, this synthetic compound promotes specific killing of breast-cancer cells via *CLs* depletion, which is at the basis of the induced michondrion-dependent apoptotic response [59, 60]. The last observation indicates that ATRA and the arylurea-fatty-acid are characterized by complementary mechanisms of anti-tumor action involving indirect or direct effects on *CLs*.

## Conclusions

Overall, the study provides new insights into the mechanisms underlying the anti-tumor activity of ATRA in breast cancer. To the best of our knowledge, this is the first study which demonstrates that the anti-proliferative effects of the retinoid are, at least partially, the consequence of a decrease in the amounts of mitochondria which leads to deficits in the respiration and energy balance of the neoplastic cells. From a basic point of view, our integrated approach defines the constitutive lipidomic profiles of luminal and basal breast-cancer cells. In addition, it demonstrates that the anti-proliferative effects of ATRA are, at least partially, the consequence of a decrease in the amounts of mitochondria which leads to deficits in the energy balance of the breast-cancer cell. As for the applicative aspect, the study opens the way to therapeutic strategies based on rational combinations involving ATRA and organic molecules targeting the mitochondria, an emerging group of anti-tumor agents.

## Supplementary information

**Additional file 1: Figures S1-S12.** Supplementary Information, Supplementary Methods, Supplementary References.

**Additional file 2: Table S1.** Lipidomics raw data, Highthroughput lipid ID.

**Additional file 3: Table S2.** Effects of ATRA on cardiolipin amounts in *SK-BR-3* cells.

**Additional file 4: Table S3.** Effects of ATRA on cardiolipin amounts in genetically engineered breast cancer *MDA-MB-453* and *SK-BR-3* cells.

## Data Availability

All the data reported by the manuscript are publicly available and the materials are also freely available upon request to the corresponding author (e-mail: enrico.garattini@marionegri.it).

## Abbreviations

APL: Acute-promyelocytic-leukemia
ATRA: All-trans-retinoic-acid
CAR: Acylcarnitines
CER: Ceramides
CL: Cardiolipins
DG: Diacylglycerols
DHCER: Dihydroceramides
ER: Estrogen-receptor
FA: Fatty acids
LBPA/BMP: Lysobisphosphatidic acid/ bis(monoacylglycero)phosphate
LPC: Lysophosphatidylcholines
LPE: Lysophosphatidylethanolamines
N-glyco-SP: Neutral glycosphingolipids
o-LPC/p-LPC: 1-alkyl-glycerophosphocholines/1-alkenyl-glycerophosphocholines
o-PC/p-PC: 1-alkyl-2-acylglycerophosphocholines/1-alkenyl-2-acylglycerophosphocholines
o-TG/p-TG: alkyldiacylglycerols/1Z-alkenyldiacylglycerols
PC: Phosphatidylcholines
PCA: Principal component analysis
PE: Phosphatidylethanolamines
PG: Phosphatidylglycerol
PI: Phosphatidylinositols
p-PE: 1-alkyl-2-acylglycerophosphoethanolamines/1-alkenyl-2-acylglycerophosphoethanolamines
PR: Progesterone-receptor
PS: Phosphatidylserines
SE: Steryl esters
SM: Sphingomyelins
SPH/SP: Sphingosines/sphinganines
TG: Triacylglycerols
