# Correction to: Role of mitochondria and cardiolipins in growth inhibition of breast cancer cells by retinoic acid

**DOI:** 10.1186/s13046-019-1485-4

**Published:** 2019-12-18

**Authors:** Mineko Terao, Laura Goracci, Valentina Celestini, Mami Kurosaki, Marco Bolis, Alessandra Di Veroli, Arianna Vallerga, Maddalena Fratelli, Monica Lupi, Alessandro Corbelli, Fabio Fiordaliso, Maurizio Gianni, Gabriela Paroni, Adriana Zanetti, Gabriele Cruciani, Enrico Garattini

**Affiliations:** 1 Laboratory of Molecular Biology, Istituto di Ricerche Farmacologiche Mario Negri IRCCS, via La Masa 19, 20156 Milan, Italy; 2 Department of Chemistry, Biology and Biotechnology, University of Perugia, via Elce di Sotto 8, 06123 Perugia, Italy; 3 Consortium for Computational Molecular and Materials Sciences (CMS), via Elce di Sotto 8, 06123 Perugia, Italy; 4 Department of Oncology, Istituto di Ricerche Farmacologiche Mario Negri IRCCS, via La Masa 19, 20156 Milan, Italy; 5 Department of Cardiovascular Research, Istituto di Ricerche Farmacologiche Mario Negri IRCCS, via La Masa 19, 20156 Milan, Italy

**Correction to: J Exp Clin Cancer Res (2019) 38:436**

**https://doi.org/10.1186/s13046-019-1438-y**

In the original publication of this article [1], the images of Figs. 4 and 5 were exchanged and the legends of the two figures did not correspond due to a typesetting error.

**Fig. 4:**
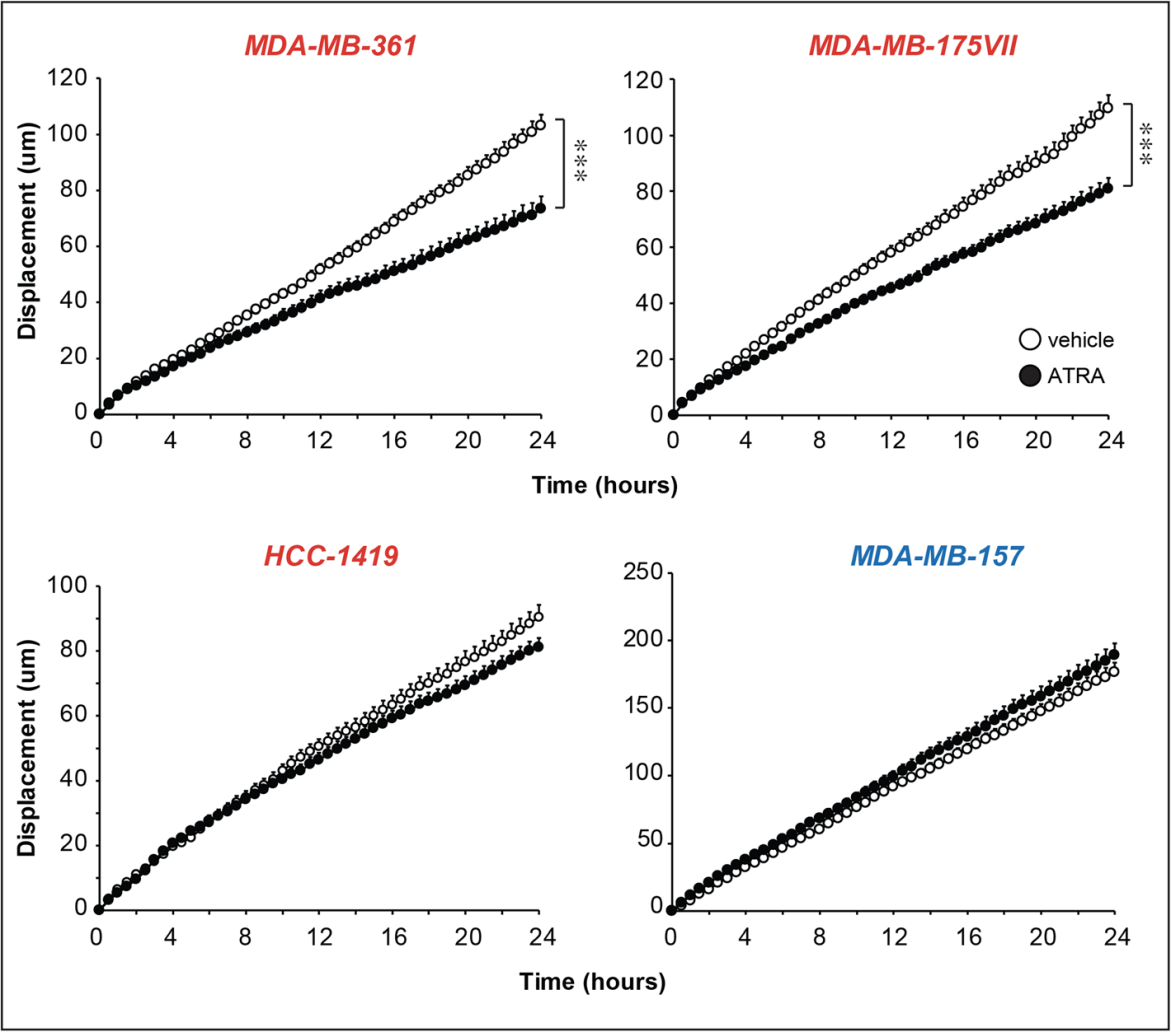
Effect of ATRA on the random motility of breast cancer cells. Biological triplicates of the indicated luminal (*MDA-MB-361*, *MDA-MB-175VII* and *HCC-1419*; marked in red) and basal (*MDA-MB-157*; marked in blue) cell lines. Cells were pre-treated with vehicle (DMSO) or ATRA. Each point is the Mean + SD of 40 cells. ***Significantly lower than the vehicle curve (*p* < 0.001 following two-way ANOVA Bonferroni post-test)

**Fig. 5:**
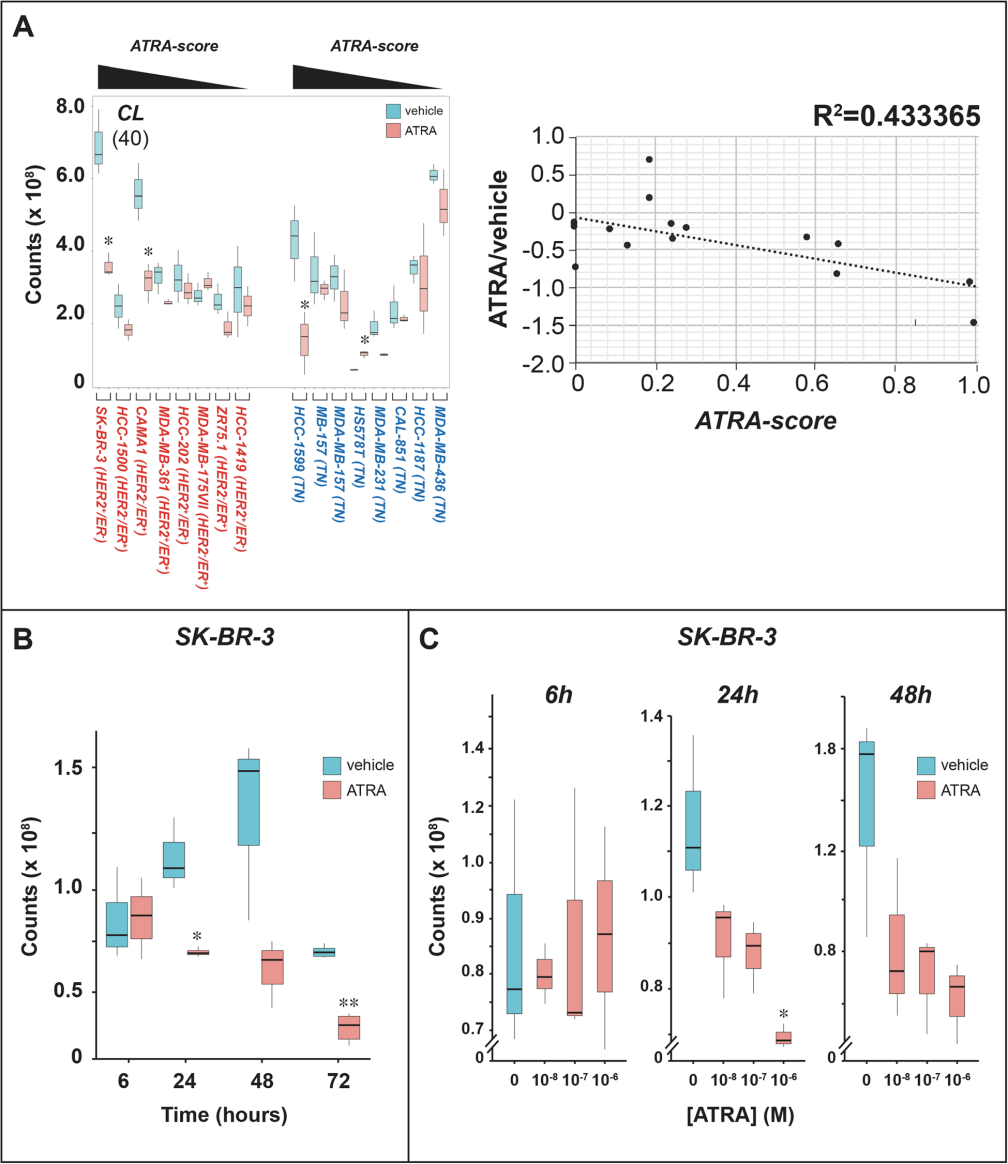
ATRA effects on the levels of cardiolipins. **a** Biological triplicates of the indicated breast cancer cells were treated with vehicle (DMSO) or ATRA (10^- 6^ M) for 48 h. Left: The box plots show the median ± SD levels of cardiolipins (*CLs*). The number of different *CL* molecules identified by mass-spectrometry is indicated in parenthesis. Luminal cell-lines are marked in red and basal cell-lines are marked in blue. The luminal and basal cell-lines are ordered according to decreasing sensitivity to the anti-proliferative effect of ATRA from left to right, as indicated (decreasing *ATRA-score*). Right: The diagram indicates the correlations between the ATRA/DMSO ratio of the mean values calculated for CLs in each cell-line and the corresponding *ATRA-score*. **b** Biological triplicates of SK-BR-3 cells were treated with vehicle (DMSO) or ATRA (10^- 6^ M) for the indicated amounts of time. The box plot shows the median ± SD levels of cardiolipins (*CLs*). **c** Biological triplicates of *SK-BR-3* cells were treated with vehicle (DMSO) or the indicated concentrations of ATRA for 48 h. The box plot shows the median ± SD levels of cardiolipins (*CLs*). *Significantly different (*p* < 0.05) from the corresponding vehicle treated control using the Student’s t-test. **Significantly different (*p* < 0.01) from the corresponding vehicle treated control using the Student’s t-test

The publisher sincerely apologizes for the inconvenience caused to the readers.

The original article has been corrected.
