# Supplementary material for: Role of mitochondria and cardiolipins in growth inhibition of breast cancer cells by retinoic acid
Source: J Exp Clin Cancer Res. 2019 Oct 29;38:436. doi: 10.1186/s13046-019-1438-y (PMC6821005; doi:10.1186/s13046-019-1438-y)
Supplement: Supplementary file 1 — Additional file 1: Figures S1-S12. Supplementary Information, Supplementary Methods, Supplementary References. [file 13046_2019_1438_MOESM1_ESM.docx]

Additional file 1

**Title:** Role of mitochondria and cardiolipins in growth inhibition of breast cancer cells by retinoic acid

Mineko Terao1^, Laura Goracci^2,3^^, Valentina Celestini1^, Mami Kurosaki^1^, Marco Bolis^1^, Alessandra Di Veroli^2^, Arianna Vallerga^1^, Maddalena Fratelli^1^, Monica Lupi^3^, Alessandro Corbelli^4^, Fabio Fiordaliso^4^, Maurizio Gianni’^1^, Gabriela Paroni^1^, Adriana Zanetti^1^, Gabriele Cruciani^2,3^ and Enrico Garattini^1^*

^1^Laboratory of Molecular Biology, Istituto di Ricerche Farmacologiche Mario Negri IRCCS, via La Masa 19, 20156 Milano, Italy.

^2^Department of Chemistry, Biology and Biotechnology, University of Perugia, via Elce di Sotto 8, 06123 Perugia, Italy.

^3^Consortium for Computational Molecular and Materials Sciences (CMS), via Elce di Sotto 8, 06123 Perugia, Italy.

^The three authors equally contributed to the generation of the data and share the first-authorship

*Correspondance should be addressed to: Enrico Garattini, Laboratory of Molecular Biology, Istituto di Ricerche Farmacologiche Mario Negri IRCCS, via La Masa 19, 20156 Milano, Italy. E-mail: enrico.garattini@marionegri.it; Tel No. +390239014533.

Supplementary Methods: Page 2-4

Supplementary References: Page 4

Supplementary Figures S1-S11: Page 5-15

SUPPLEMENTARY METHODS

*Cell lines*

The table below summarizes the characteristics and the source of the cell lines used throughout the study: ATCC = Americam Type Culture Collection; DSMZ = Deutsche Sammlung von Mikroorganismen und Zellkulturen; SIGMA = Sigma-Aldrich.

*Untargeted lipidomics analysis*

Untargeted lipidomics analysis was performed by using Lipostar, a vendor-neutral highthroughput software to support targeted and untargeted LC-MS lipidomics [1]. The major innovative points in the Lipostar algorithms are the matrix-based procedure for isotopes and adducts handling, the lipid identification module (with or without the use of a lipid fragment database) and a series of multivariate statistical analysis tools (supervised and unsupervised). In this work, raw data files acquired in Full Scan mode were uploaded in Lipostar to generate the data matrix. Thus, a preliminary untargeted lipid identification run was carried out based on a database of fragmented lipids (approximately 850.000 compounds), using a tolerance of 5 ppm for m/z values and using a pre-defined range of RT values for each lipid class and subclass. Afterwards, the features identified as potential lipids were used to generate an inclusion list to acquire MS/MS data of interest in a second LC-MS run. Once MS/MS were available, they were imported in the Lipostar session and used to run the final lipid identification based on m/z, RT and MS/MS information. At the end of the process, the lipid profile automatically generated by Lipostar was refined by visual inspection to generate the final lipid profile composed of 530 chemical features from lipid species in one or multiple adduct forms. To compare lipid profiles, the average lipid profile for each sample was obtained from replicates. The Statistical analysis module in Lipostar was used to perform Principal Component Analysis (PCA), with data treated by applying the Pareto scaling. Similar types of analyses were performed in the experiments involving the RARα over-expressing and RARα silenced cell-lines.

*Lipid extraction and sample preparation*

Lipids from cells samples were extracted in a methanol:MTBE:chloroform (MMC) mixture (40/30/30, v/v/v) [2], containing the antioxidant BHT (10μg/100mL). Cells pellets were extracted

adding to each sample the opportune volume of MMC based on cells number (2.5x106 cells/1.0 ml). Each sample was vortexed and placed in a shaker at room temperature for 30 minutes (950 rpm). Samples were centrifuged 10 minutes at 8,000 rpm and the supernatant saved for analysis in fresh Eppendorf tubes. These experimental conditions were applied to all the cell-lines with the exception of *HCC-1599* cells. In the case of this last cell-line, the number of cells subjected to the extraction procedure was (10 x 10^6^ cells/1.0 ml), as *HCC-1599* grow in suspension. Nevertheless, the raw data on the levels of the lipids identified in *HCC-1599* cells (Supplementary Table S1) were normalized by multiplying them by 0.25.

*Mass-spectrometry*

A volume of 2 μL of each sample was injected onto the LC-MS system. The LC-MS system

consisted of a Binary pump, thermostated autosampler, and column compartment, all Dionex Ulimate

3000 series modules (Thermo Fisher Scientific, Waltham, MA USA) and a Thermo Q-exactive mass

spectrometer (Thermo Fisher Scientific, Waltham, MA USA). Liquid chromatography separation was

performed at 45°C using a reverse phase column, Kinetex F5 (Phenomenex inc.), at a flow rate of 0.65 ml/min. The mobile phases consisted of 5 mM ammonium formate and 0.1% formic acid in water and 5 mM ammonium formate and 0.1% formic acid in isopropanol. A gradient elution was used for the lipid separation as follows: time 0 min, solvent A 80%, B 20%; time 3 min, solvent A 60%, B 40%; time 16 min, solvent A 40%, B 60%; time 16.5 min, solvent A 30%, B 70%; time 24 min, solvent A 26%, B 74%; time 28 min, solvent A 5%, B 95%; and time 30, stop run. All solvents were purchased from Sigma-Aldrich and Biosolve (Dieuze, FR). Mass spectrometry analysis was first performed with both positive/negative ion switching methods in Full MS scan mode. The Lipostar software [1] was used to perform a pre-identification of potential lipid species based on m/z and RT values, and to generate the corresponding inclusion list of masses of interest. Thus, a reduced number of samples automatically selected by Lipostar to assure the coverage of the entire inclusion list was analyzed again in DDS mode through the use of the inclusion list to get MSMS data. The workflow established for these experiments was followed by statistical analysis and identification process for each compound.

SUPPLEMENTARY REFERENCES

1. Goracci L, Tortorella S, Tiberi P, Pellegrino RM, Di Veroli A, Valeri A, Cruciani G (2017) Lipostar, a Comprehensive Platform-Neutral Cheminformatics Tool for Lipidomics. Anal Chem 89: 6257–6264.
2. Pellegrino RM, Di Veroli A, Valeri A, Goracci L, Cruciani G (2014) LC/MS lipid profiling from human serum: a new method for global lipid extraction. Anal Bioanal Chem 406: 7937–7948.
3. Salmona M, Forloni G, Diomede L, Algeri M, De Gioia L, Angeretti N, Giaccone G, Tagliavini F, Bugiani O. A neurotoxic and gliotrophic fragment of the prion protein increases plasma membrane microviscosity. Neurobiol Dis. 1997; 4: 47–57.

**
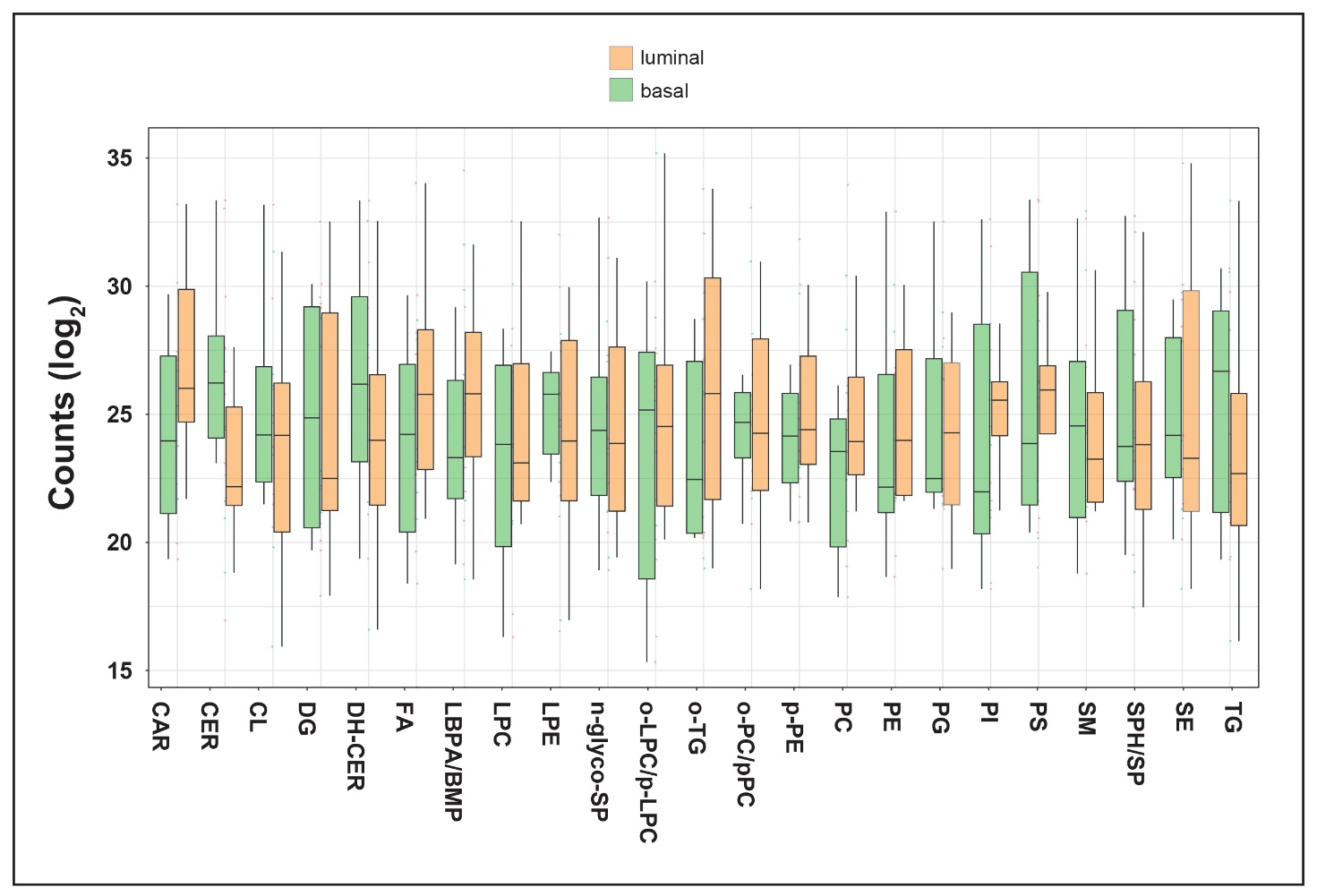
**

**Figure S1** *Differences in the levels of the identified classes of lipids in luminal and basal cell-lines*

The box plots show the median + SD levels of the indicated lipid classes observed in the 8 basal cell-lines (*HCC-1599*, *MB-157*, *MDA-MB-157*, *HS578T*, *MDA-MB-231*, *CAL-851*, *HCC-1187* and *MDA-MB-436*) and the 8 luminal (*SK-BR-3*, *HCC-1500*, *CAMA1*, *MDA-MB-361*, *HCC-202*, *MDA-MB-175VII*, *ZR75.1* and *HCC-1419*) cell-lines of the panel.

**
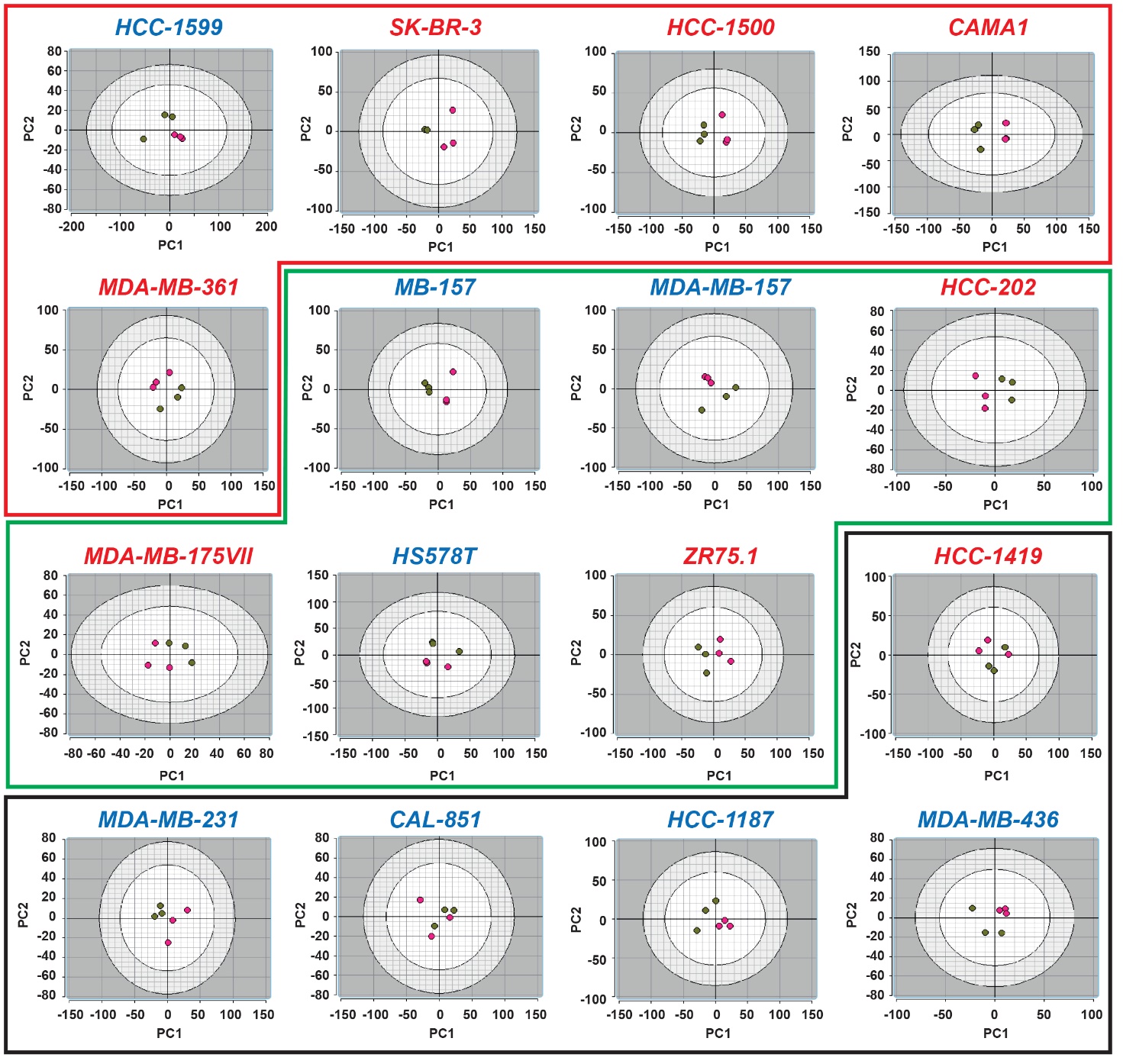
**

**Figure S2** *Effects of ATRA on the lipidomic profiles of breast cancer cells*

Biological triplicates of the indicated breast cancer cells were treated with vehicle (DMSO) or ATRA (10^-6^ M) for 48 hours. The diagrams indicate bidimensional primary component analysis of the lipidomic data obtained. Green points = vehicle; pink points = ATRA. Basal cell lines are marked in blue and luminal cell lines are marked in red. The cell lines are ordered according to a decreasing *ATRA-score* from left to right. Cell lines characterized by high (high sensitivity to ATRA), intermediate (intermediate sensitivity to ATRA) and low (low-sensitivity/resistance to ATRA) *ATRA-score* values are contained within red, green and black squares, respectively.

**
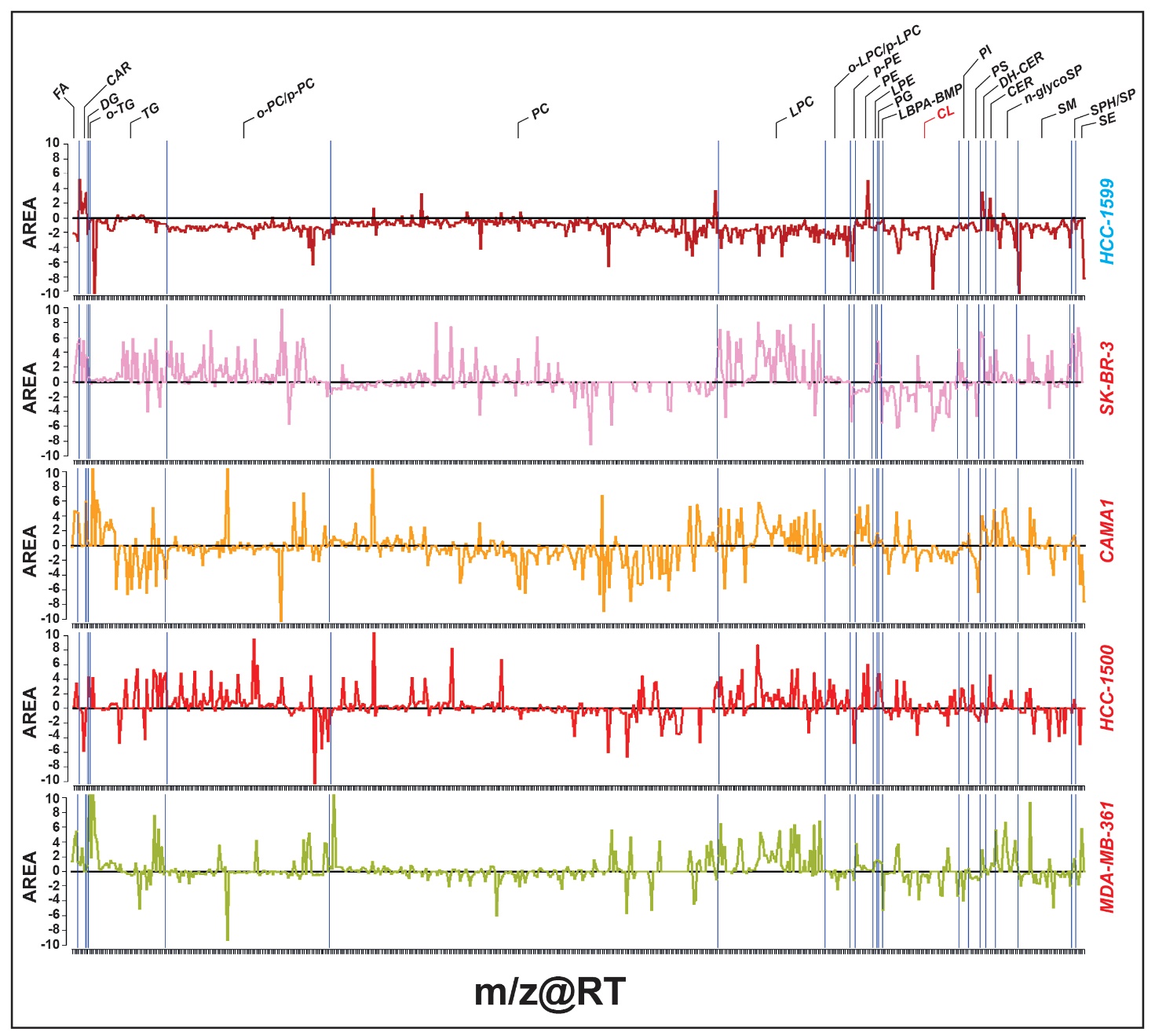
**

**Figure S3** *ATRA effects on the lipidomic profiles of breast cancer cells characterized by a high ATRA-score*

Biological triplicates of the indicated breast cancer cells characterized by high retinoid sensitivity (high *ATRA-score*) were treated with vehicle (DMSO) or ATRA (10^-6^ M) for 48 hours. The diagrams show the effects of ATRA on the single lipids identified by mass-spectrometric analysis and grouped in homogeneous classes. Basal cell lines are marked in blue and luminal cell lines are marked in red. The cell lines are ordered according to a decreasing *ATRA-score* from top to bottom. *FA*=fatty acids; *CAR*=acylcarnitines; *DG*=diacylglycerols; *o-TG/pTG*=alkyldiacylglycerols/1Z-alkenyldiacylglycerols; *TG*=triacylglycerols; *o-PC/p-PC*=1-alkyl-2acylglycerophosphocholines/1-alkenyl-2-acylglycerophosphocholines; *PC*=phosphatidylcholines; *LPC*=lysophosphatidylcholines; *o-LPC/p-LPC*=1-alkyl-glycerophosphocholines/1-alkenylglycerophosphocholines; *p-PE*=1-alkyl-2-acylglycerophosphoethanolamines/1-alkenyl-2acylglycerophosphoethanolamines; *PE*=phosphatidylethanolamines; *LPE*=lysophosphatidylethanolamines; *PG*=phosphatidylglycerol; *LBPA/BMP*=lysobisphosphatidic acid/bis(monoacylglycero)phosphate; *CL*=cardiolipins; *PI*=phosphatidylinositols; *PS*=phosphatidylserines; *DH-CER*=dihydroceramides; *CER*=ceramides; *N-glyco-SP*=neutral glycosphingolipids; *SM*=sphingomyelin; *SPH/SP*=sphingosines/sphinganines; *SE*=steryl esters. Cardiolipins (*CL* are marked in red).

**
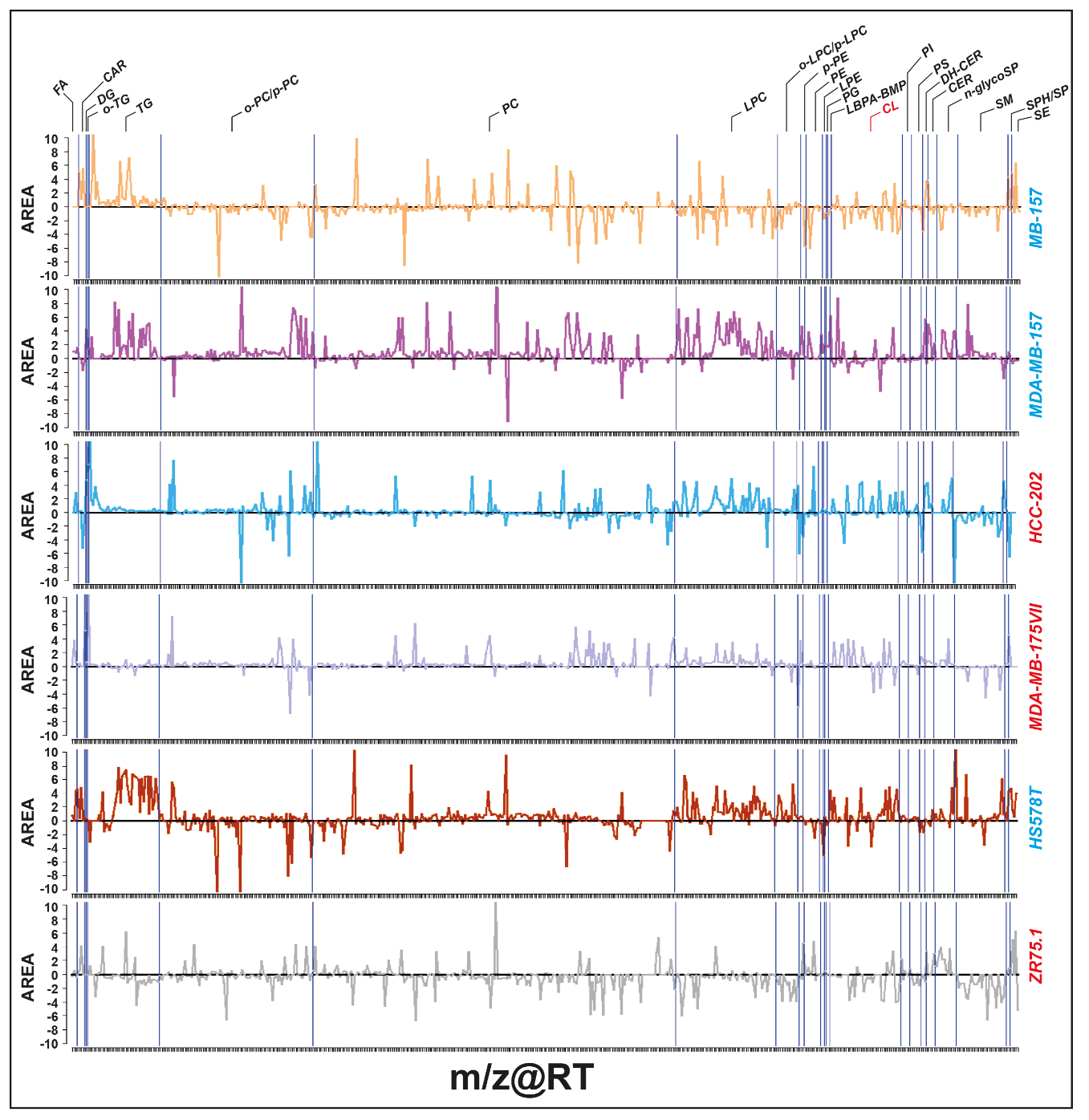
**

**Figure S4** *ATRA effects on the lipidomic profiles of breast cancer cells characterized by an intermediate ATRA-score*

Biological triplicates of the indicated breast cancer cells characterized by intermediate retinoid sensitivity (low *ATRA-score*) were treated with vehicle (DMSO) or ATRA (10^-6^ M) for 48 hours. The diagrams show the effects of ATRA on the single lipids identified by massspectrometric analysis and grouped in homogeneous classes. Basal cell lines are marked in blue and luminal cell lines are marked in red. The cell lines are ordered according to a decreasing *ATRA-score* from top to bottom. *FA*=fatty acids; *CAR*=acylcarnitines; *DG*=diacylglycerols; *o-TG/pTG*=alkyldiacylglycerols/1Z-alkenyldiacylglycerols; *TG*=triacylglycerols; *o-PC/p-PC*=1-alkyl-2acylglycerophosphocholines/1-alkenyl-2-acylglycerophosphocholines; *PC*=phosphatidylcholines; *LPC*=lysophosphatidylcholines; *o-LPC/p-LPC*=1-alkyl-glycerophosphocholines/1-alkenylglycerophosphocholines; *p-PE*=1-alkyl-2-acylglycerophosphoethanolamines/1-alkenyl-2acylglycerophosphoethanolamines; *PE*=phosphatidylethanolamines; *LPE*=lysophosphatidylethanolamines; *PG*=phosphatidylglycerol; *LBPA/BMP*=lysobisphosphatidic acid/bis(monoacylglycero)phosphate; *CL*=cardiolipins; *PI*=phosphatidylinositols; *PS*=phosphatidylserines; *DH-CER*=dihydroceramides; *CER*=ceramides; *N-glyco-SP*=neutral glycosphingolipids; *SM*=sphingomyelin; *SPH/SP*=sphingosines/sphinganines; *SE*=steryl esters. Cardiolipins (*CL* are marked in red).

**
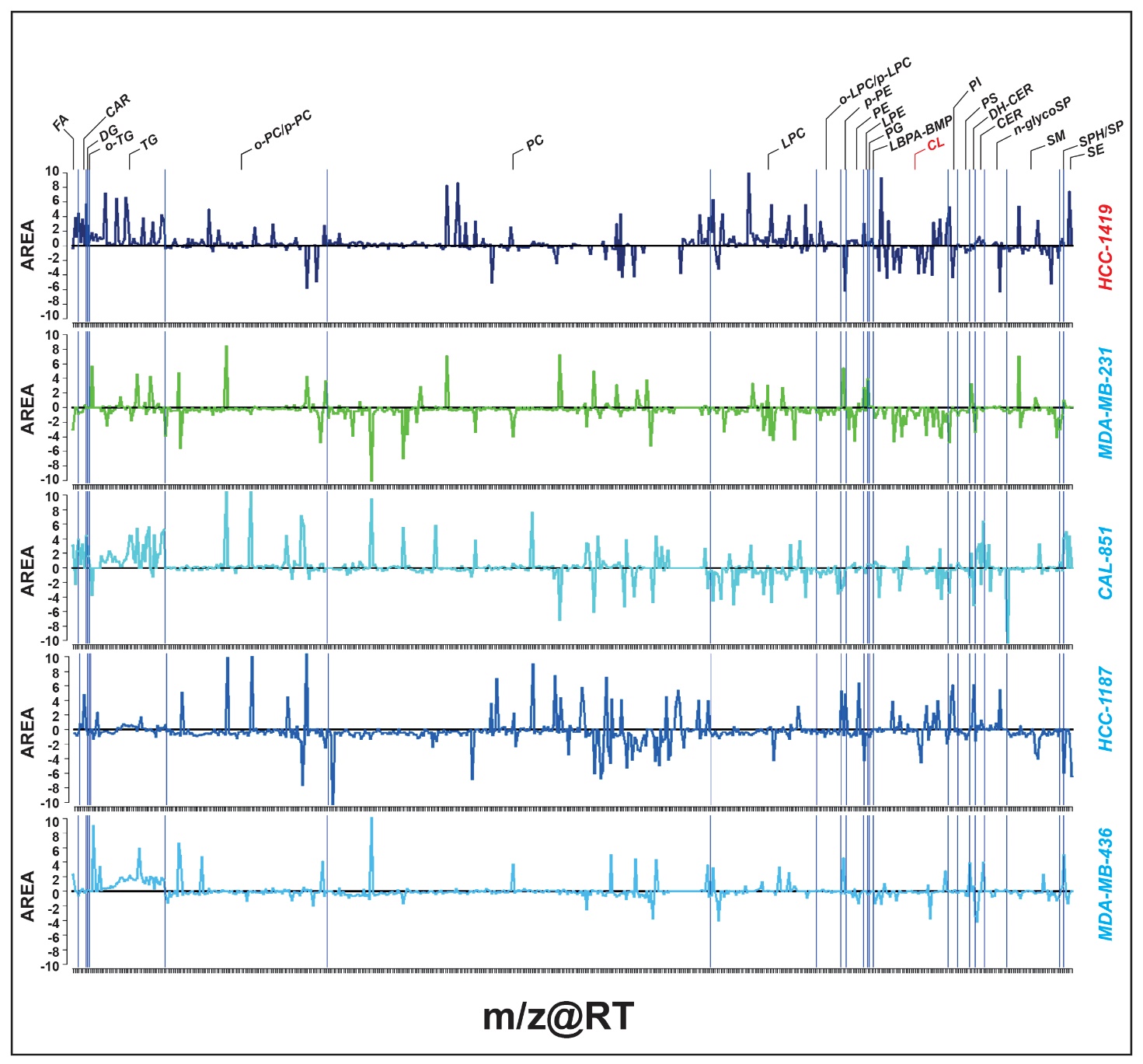
**

**Figure S5** *ATRA effects on the lipidomic profiles of breast cancer cells characterized by a low ATRA-score*

Biological triplicates of the indicated breast cancer cells characterized by low retinoid sensitivity (low *ATRA-score*) were treated with vehicle (DMSO) or ATRA (1 µM) for 48 hours. The diagrams show the effects of ATRA on the single lipids identified by mass-spectrometric analysis and grouped in homogeneous classes. Basal cell lines are marked in blue and luminal cell lines are marked in red. The cell lines are ordered according to a decreasing *ATRA-score* from top to bottom. *FA*=fatty acids; *CAR*=acylcarnitines; *DG*=diacylglycerols; *o-TG/pTG*=alkyldiacylglycerols/1Z-alkenyldiacylglycerols; *TG*=triacylglycerols; *o-PC/p-PC*=1-alkyl-2acylglycerophosphocholines/1-alkenyl-2-acylglycerophosphocholines; *PC*=phosphatidylcholines; *LPC*=lysophosphatidylcholines; *o-LPC/p-LPC*=1-alkyl-glycerophosphocholines/1-alkenylglycerophosphocholines; *p-PE*=1-alkyl-2-acylglycerophosphoethanolamines/1-alkenyl-2acylglycerophosphoethanolamines; *PE*=phosphatidylethanolamines; *LPE*=lysophosphatidylethanolamines; *PG*=phosphatidylglycerol; *LBPA/BMP*=lysobisphosphatidic acid/bis(monoacylglycero)phosphate; *CL*=cardiolipins; *PI*=phosphatidylinositols; *PS*=phosphatidylserines; *DH-CER*=dihydroceramides; *CER*=ceramides; *N-glyco-SP*=neutral glycosphingolipids; *SM*=sphingomyelin; *SPH/SP*=sphingosines/sphinganines; *SE*=steryl esters. Cardiolipins (*CL* are marked in red).

**
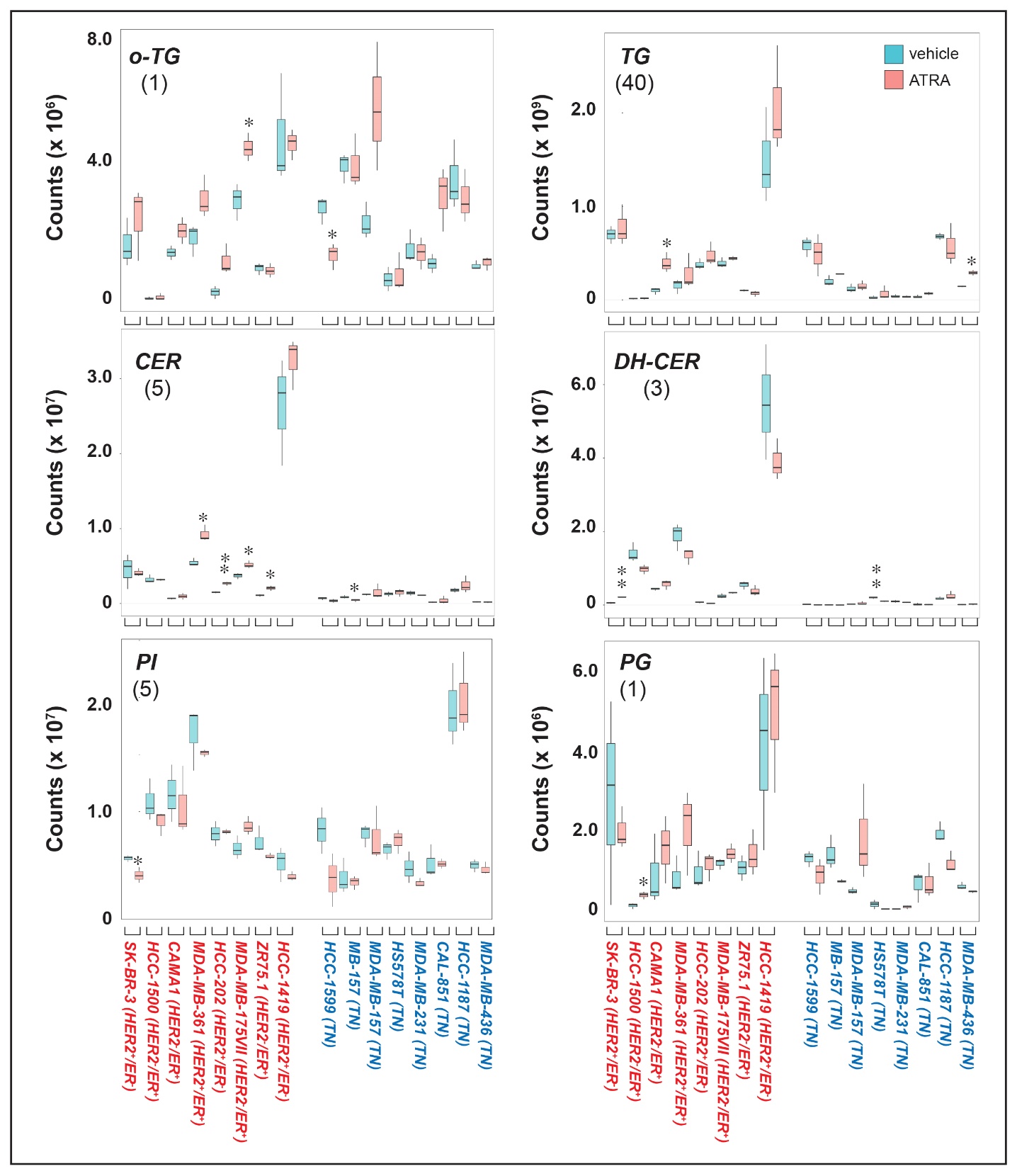
**

**Figure S6** *Effects of ATRA on specific lipid classes in breast cancer cells*

Biological triplicates of the indicated breast cancer cells were treated with vehicle (DMSO) or ATRA (10^-6^ M) for 48 hours. The box plots show the median + SD levels of alkyldiacylglycerols/1Z alkenyldiacylglycerols (*o-TG/p-TG*), triacylglycerols (*TG*), ceramides (*CER*), dihydroceramides (*DHCER*), phosphatidylinositols (*PI*) and phosphatidylglycerol (*PG*). The number of different molecules identified by mass-spectrometry is indicated in parenthesis. Basal cell-lines are marked in blue and luminal cell-lines are marked in red. The luminal and basal cell-lines are ordered according to decreasing sensitivity to the anti-proliferative effect of ATRA from left to right, as indicated (decreasing *ATRA-score*).

**
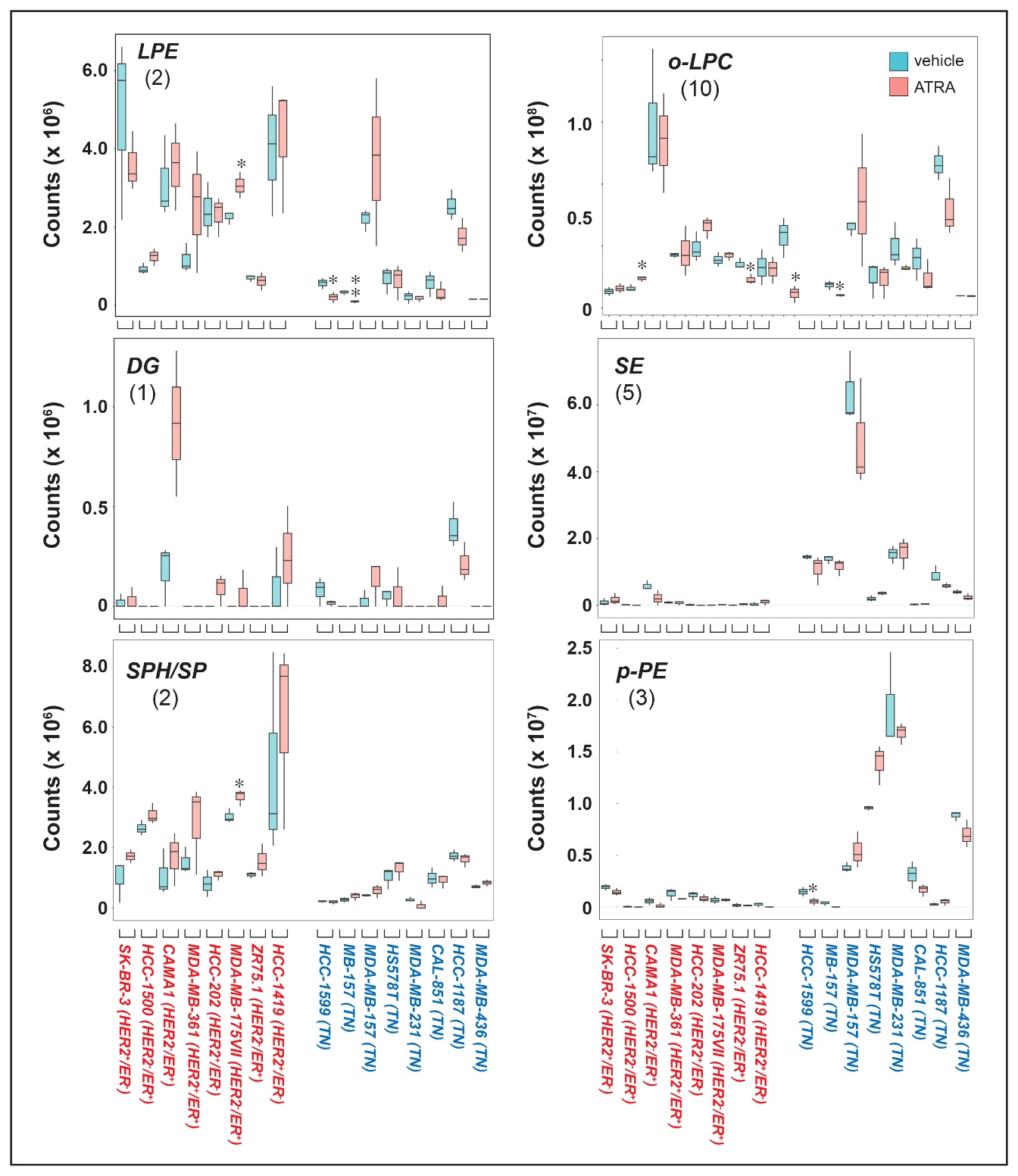
**

**Figure S7** *Effects of ATRA on specific lipid classes in breast cancer cells*

Biological triplicates of the indicated breast cancer cells were treated with vehicle (DMSO) or ATRA (10^-6^ M) for 48 hours. The box plots show the median + SD levels of lysophosphatidylethanolamines (LPE), 1-alkyl-glycerophosphocholines/1-alkenyl glycerophosphocholines (*o-LPC*), diacylglycerols (*DG*), steryl esters (*SE*), sphingosines/sphinganines (*SPH/SP*) and 1-alkyl-2-acylglycerophosphoethanolamines/1-alkenyl-2-acylglycerophosphoethanolamines (*p-PE*). The number of different molecules identified by mass-spectrometry is indicated in parenthesis. Basal cell-lines are marked in blue and luminal cell-lines are marked in red. The luminal and basal cell-lines are ordered according to decreasing sensitivity to the anti-proliferative effect of ATRA from left to right, as indicated (decreasing *ATRA-score*). *Significantly different (p<0.05) from the corresponding vehicle treated control using using the Student’s t-test. **Significantly different (p<0.01) from the corresponding vehicle treated control using using the Student’s t-test.

**
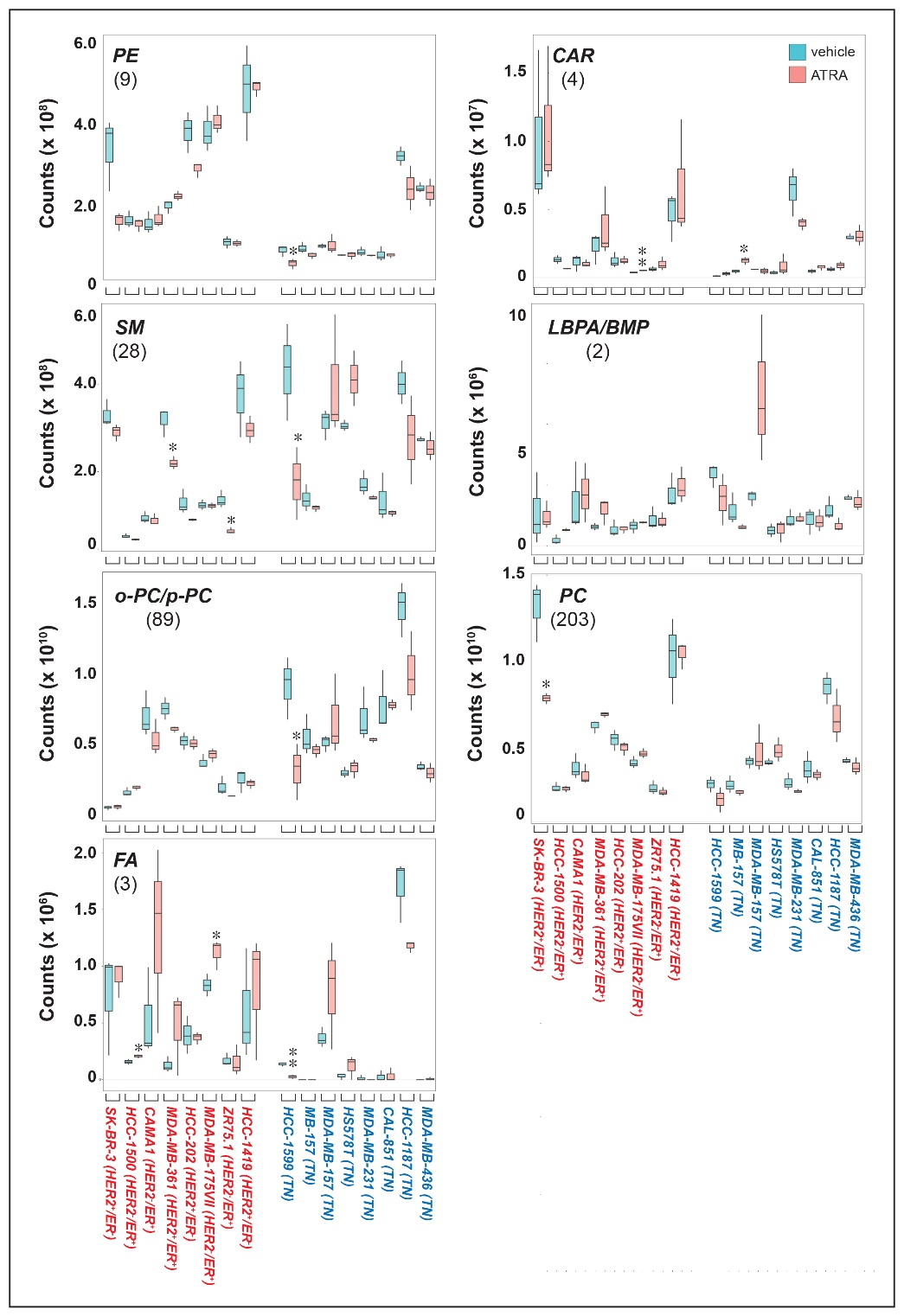
**

**Figure S8** *Effects of ATRA on specific lipid classes in breast cancer cells*

Biological triplicates of the indicated breast cancer cells were treated with vehicle (DMSO) or ATRA

(10^-6^ M) for 48 hours. The box plots show the median + SD levels of phosphatidylethanolamines (*PE*), acylcarnitines (*CAR*), sphingomyelin (*SM*), lysobisphosphatidicacid/bis(monoacylglycero)phosphate (*LBPA* or *BMP*), 1-alkyl-2-acylglycerophosphocholines/1-alkenyl-2-acylglycerophosphocholines (*o-PC/p-PC*), phosphatidylcholines (*PC*) and fatty acids (*FA*). The number of different molecules identified by mass-spectrometry is indicated in parenthesis. Basal cell-lines are marked in blue and luminal cell-lines are marked in red. The luminal and basal cell-lines are ordered according to decreasing sensitivity to the anti-proliferative effect of ATRA from left to right, as indicated (decreasing *ATRA-score*). *Significantly different (p<0.05) from the corresponding vehicle treated control using using the Student’s t-test. **Significantly different (p<0.01) from the corresponding vehicle treated control using using the Student’s t-test.

**
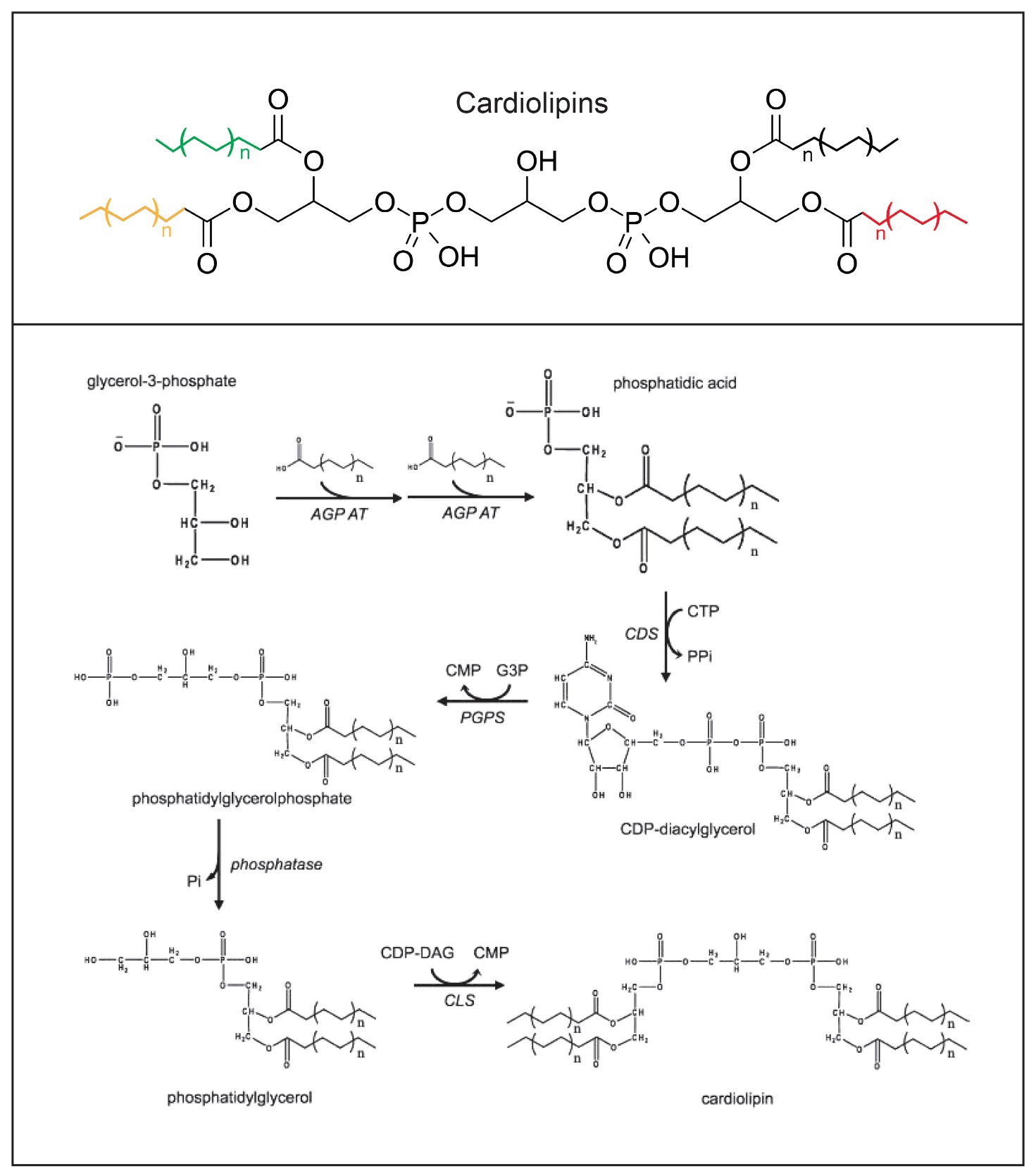
**

**Figure S9** *Structure and biosynthesis of cardiolipins*

Upper: The panel illustrates the chemical structure of cardiolipins. Lower: The simplified scheme (https://en.wikipedia.org/wiki/Cardiolipin) shows the structure and the last steps of the cardiolipins biosynthetic pathway. AGPAT = 1-acylglycerol-3-phosphate O-acyltransferase (6 known isoforms: AGPAT1, AGPAT2, AGPAT3, AGPAT4 and AGPAT5); CDS = CDP-diacylglycerol synthase (2 known isoforms: CDS1 and CDS2); PGS1 = phosphatidylglycerophosphate synthase 1; PTPMT1 = protein tyrosine phosphatase mitochondrial 1; CRLS1 = cardiolipin synthetase1.

**
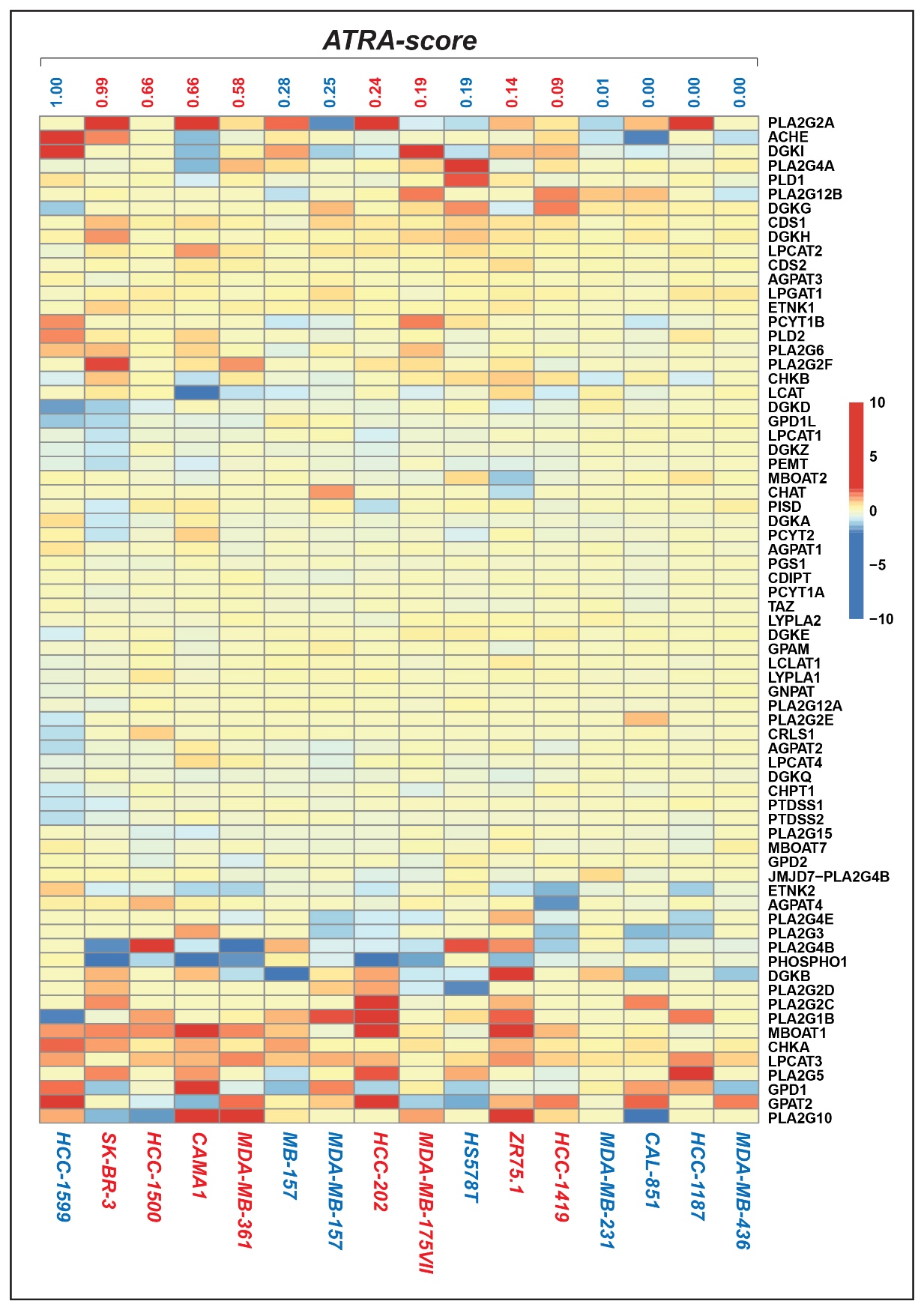
**

**Figure S10** *ATRA effects on the expression of genes involved in the biosynthesis/metabolism of glycerophospholipids*

Biological triplicates of the indicated breast cancer cells treated with vehicle (DMSO) or ATRA (10^-6^ M) for 24 hours. Total RNA was extracted and subjected to RNA-seq analysis. The heat-map shows the expression profiles of the genes belonging to the “Glycerophospholipids” metabolic pathway (https://www.genome.jp/kegg/pathway.html). The results are expressed in log_2_ values of the ATRA/DMSO ratio as indicated. The *ATRA-score* of each cell-line is indicated above the heat-map and the cell-lines are ordered according to their decreasing sensitivity to ATRA from left to right.

**
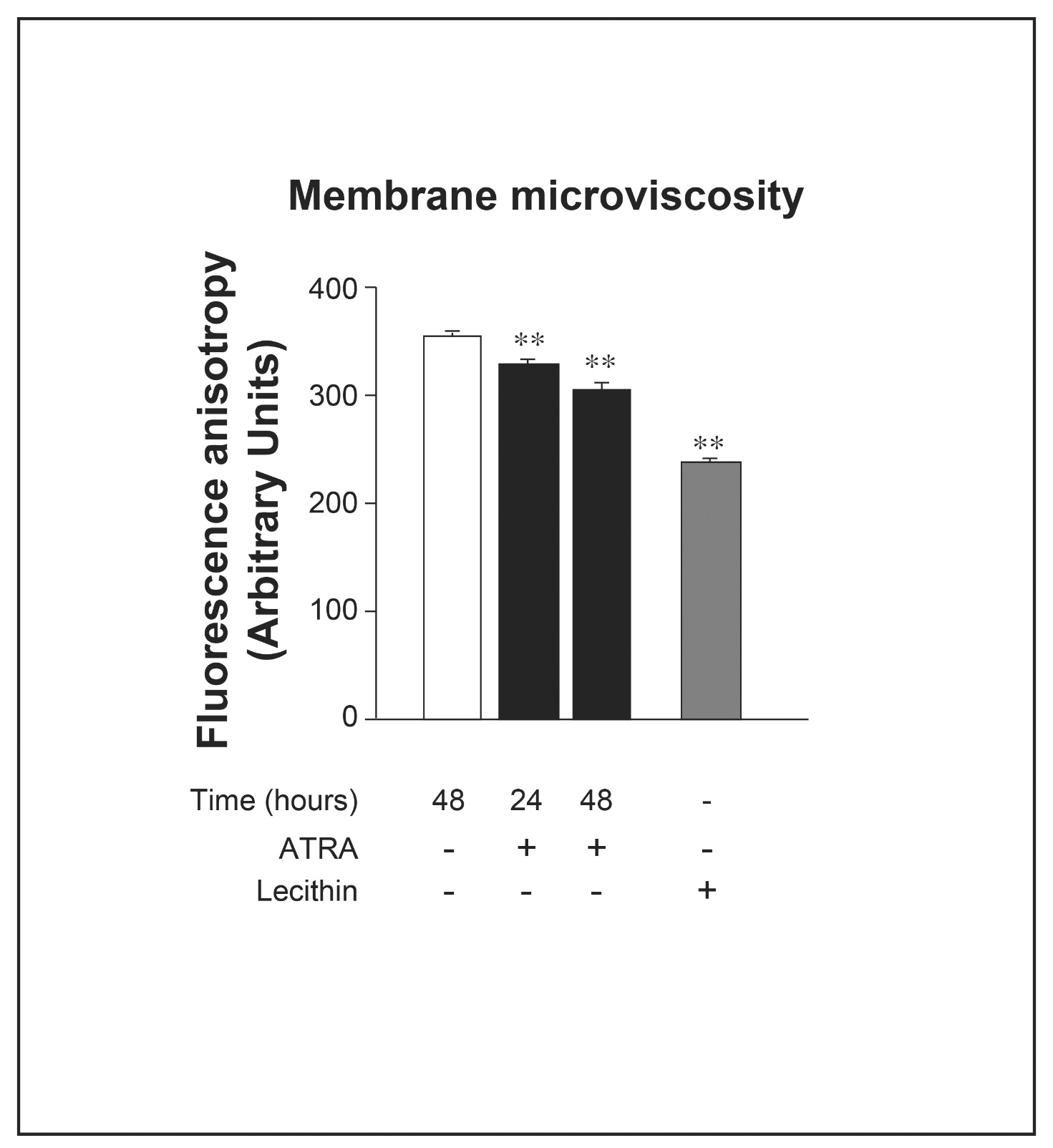
**

**Figure S11** *ATRA effects on mitochondrial membrane microviscosity*

Three replicate cultures of *SK-BR-3* cells were treated with vehicle (DMSO) or ATRA (10^-6^ M) for the indicated amount of time. At the end of the treatment, mitochondria were isolated and incubated with 1,6-diphenyl-1,3,5-hexatriene to assess the microviscosity of mitochondrial membranes. Mitochondria incubated with egg yolk lecithin, which is a membrane fluidifier, were used as an internal positive control for the experiment. The values are expressed as the Mean+SD of the membrane microviscosity values (N = 3). ** Significantly lower than the DMSO treated control (p<0.01, Student’s t-test).
